# Bio-mineralization process of CaCO_3_ induced by bacteria isolated from Egypt for sustainable bio-concrete

**DOI:** 10.1186/s12934-025-02837-3

**Published:** 2025-09-16

**Authors:** Dalia Said, Sahar M. Ibrahim, Mohamed Heikal, Mohamed O. Abdel-Monem, Ghada E. Dawwam

**Affiliations:** 1 Botany and Microbiology Department, Faculty of Science, Benha University, Benha, Egypt; 2 Chemistry Department, Faculty of Science, Benha University, Benha, Egypt

**Keywords:** Bioconcrete, CaCO_3_ precipitation, *Bacillus tropicus*, Optimization, Characterization, Strength, Pore structure, Microstructure

## Abstract

Microbial-induced calcium carbonate precipitation (MICP) has garnered significant attention for its construction and geotechnical engineering applications. In this study, 24 bacterial isolates were obtained from various edges of Wadi El-Natron Lake, Egypt, and subsequently assessed for their capacity for calcium carbonate (CaCO₃) precipitation. Among these isolates, strain D16 exhibited the highest CaCO₃ precipitation, yielding 0.404 g/100 mL, alongside robust bacterial growth and a final pH of 9.09. Morphological and biochemical characterization revealed that the isolate was rod-shaped, Gram-positive, Catalase-positive, Urease-positive, and Spore-forming. The optimal growth conditions for the isolate included a pH of 8, with ideal Ca²⁺ and urea concentrations of 25 mM and 20 g/L, respectively, at an incubation temperature of 30 °C over seven days. Molecular identification confirmed the isolate as *Bacillus tropicus* strain D16, which has been recorded in GenBank under the accession number PQ817131. The precipitated CaCO₃ was quantified and characterized using scanning electron microscopy (SEM) equipped with energy-dispersive X-ray (EDX) analysis, Fourier-transform infrared (FT-IR) spectroscopy, X-ray diffraction (XRD), and the N₂ desorption/adsorption isotherm (BET) method. The effect of calcium carbonate nanoparticles (CaCO₃-NPs, denoted as NC) on the properties of cement paste was investigated. Four composite pastes were prepared with varying dosages of CaCO₃-NPs: NC0.0, NC0.5, NC1.0, and NC1.5. These pastes were subjected to a series of tests, including compressive strength, bulk density, total porosity, and chemically combined water content, over a hydration period of up to 90 days. The results demonstrated that the addition of NC enhanced the compressive strength of the cement paste up to an optimal dosage content of 0.5%, beyond which the strength decreased due to nanoparticle agglomeration. These findings were further corroborated by X-ray Diffraction (XRD), Differential Thermal Thermogravimetric Analysis (DTG/TGA), and Scanning Electron Microscopy (SEM), which provided microstructural and phase composition insights. Overall, the results indicate that the inclusion of an optimal dosage of CaCO₃-NPs can significantly improve the performance of cement composite pastes.

## Introduction

Concrete is among the most widely employed construction materials, esteemed for its durability, great compressive strength, widespread availability, affordability, and relatively low cost compared to alternative construction materials [1]. However, concrete remediation presents significant challenges, as conventional repair methods are often time-consuming and environmentally detrimental due to the reliance on synthetic repair agents such as resin and epoxy [2, 3]. These agents are limited in their effectiveness, as they primarily address external fractures while failing to repair microcracks or internal structural damage. Consequently, there has been an upward research interest in developing advanced, sustainable, and eco-friendly repair techniques, and smart materials that contribute to the advancement of green concrete solutions.

Vijay et al. highlighted that bacterial-based crack healing has been extensively studied as a viable approach to overcoming these challenges [4]. In bacterial-based self-healing concrete, when cracks constitute, the ingress of water activates the dormant bacterial spores, triggering their growth. This process facilitates the precipitation of minerals such as calcite (CaCO₃), effectively sealing the cracks. Furthermore, if new cracks develop under favorable environmental conditions, the bacteria can reactivate and initiate the healing process once again. Therefore, bacteria operate as a persistent self-healing agent through a process known as microbial-induced calcium carbonate precipitation (MICP) [5]. In recent years, this biomaterial has been termed bio-concrete, which is defined as concrete incorporating bacteria capable of precipitating calcite (CaCO₃), thereby exhibiting intrinsic self-healing properties [6].

Recently, bacterial-based self-healing concrete technology has been a prominent area of research, driven by the objectives of reducing repair costs, minimizing environmental impact, enhancing concrete durability and mechanical properties [7, 8], extending the lifespan of concrete structures, and decreasing resource consumption in concrete fabrication [9]. Wang et al. reported that calcium-based healing agents, such as calcium carbonate or lactate, in combination with bacteria-typically from the *Sporosarcina* or *Bacillus* genera, constitute the fundamental components of self-healing concrete [10]. These bacteria metabolize the calcium-based healing agents, leading to calcium carbonate (CaCO₃) precipitation as a metabolic byproduct [11]. This process effectively.

repairs the concrete by filling cracks, thereby restoring structural integrity [12].

Key biological variables influencing bacterial-based self-healing in concrete include the age, population density, and physiological state of bacterial cells, all of which are affected by environmental factors such as pH and temperature [13]. The high pH levels typically found in concrete contribute to prolonged crack self-healing, as certain bacterial strains can survive under such conditions by transitioning from their vegetative state into durable spores capable of withstanding extreme alkalinity [14, 15]. When bacteria are introduced into concrete cracks, they facilitate calcium carbonate formation, effectively closing cracks and improving structural integrity [16].

Ureolytic bacteria are more effective in crack healing than non-ureolytic, as the latter find it difficult to survive in the highly alkaline environment of concrete [17]. Studies have shown that non-ureolytic bacteria, such as *Bacillus thuringiensis* and *Bacillus halodurans*, can restore only up to 65% of the original strength and repair cracks up to 0.45 mm. In contrast, ureolytic bacteria, including *Bacillus subtilis*, *Sporosarcina pasteurii*, *Bacillus sphaericus*, and *Bacillus megaterium*, demonstrate superior crack-healing capabilities, effectively sealing cracks ranging from 0.85 to 0.97 mm [18].

Gram-positive, alkali-tolerant bacteria, particularly those from the Bacillus genus, are the most widely utilized microorganisms for bacterial-based self-healing concrete owing to their ability to thrive in high-pH and high-temperature environments [19, 20]. For instance, *Bacillus cereus* exhibits adaptability to extreme environmental conditions, including elevated temperatures and pH levels, contributing to reduced water permeability and effective crack repair in concrete [19]. Similarly, *Bacillus sphaericus* facilitates calcite precipitation, which enhances concrete strength and decreases water absorption. Under extreme conditions, it forms resilient endospores, ensuring long-term viability [21, 22]. Additionally, *Bacillus subtilis* demonstrates urease activity at pH 9, enabling it to tolerate harsh environments while continuously producing calcium carbonate (CaCO₃), further supporting the self-healing mechanism in concrete [23, 24].

Numerous studies have confirmed the diverse applications of calcium carbonate (CaCO₃) in environmental remediation. Eltarahony et al. successfully remediated Pb²⁺ and Hg²⁺ via CaCO₃ precipitation induced by *Proteus mirabilis* 10B under both aerobic and anaerobic nitrate-utilizing conditions [25]. Similarly, Awadeen et al. demonstrated that carbonic anhydrase (CA) enzyme facilitated the immobilization and encapsulation of Zn²⁺ and Cr⁶⁺ within a vaterite matrix through microbial biomineralization, offering an eco-friendly approach to mitigate heavy metal toxicity and limit their mobility in soil and wastewater [26]. Furthermore, Eltarahony et al. employed a microbially induced calcium carbonate precipitation (MICP) strategy for the removal of Pb²⁺ and Hg²⁺ under urea hydrolysis conditions [27].

Calcium carbonate exists in three naturally occurring polymorphs: calcite, aragonite, and vaterite. Among these, calcite is the most thermodynamically stable form and is predominantly found in marble and limestone. Most natural limestone primarily consists of calcite. Vaterite, in contrast, is the least stable and least abundant polymorph in nature. Due to its high solubility in water, vaterite readily transforms into calcite or aragonite in aqueous environments [28]. Based on particle size, calcium carbonate can be classified into three categories: macro-calcium carbonate, micro-calcium carbonate, and nano-calcium carbonate [29].

Nano-calcium carbonate (nano-CaCO₃) is widely utilized in cementitious composites due to its significant physical and chemical effects, including the filler effect and nucleation effect. However, the agglomeration of nano-CaCO₃ particles can substantially diminish these beneficial impacts [30]. The incorporation of nanoparticles into cement matrices offers various advantages, such as enhanced compressive strength, increased tensile strength and ductility, improved aggregate–paste bonding, and superior thermal resistance, making these composites suitable for use in refractory concrete applications [31].

Several studies have investigated the integration of nano-CaCO₃ into cementitious materials. In these studies, synthesized nano-CaCO₃ particles were added to cementitious composites in varying percentages based on the weight of cement. The results showed that after 7 days of curing, both flexural and compressive strengths increased with the addition of CaCO₃, with an optimal enhancement observed at approximately 2%. However, after 28 days of curing, a reduction in mechanical properties was observed in the specimens containing CaCO₃ compared to the control samples [32]. This outcome suggests that nano-CaCO₃ accelerates the hydration process by acting as a nucleation site for hydration product formation. Shaikh and Steve examined the influence of nano-CaCO₃ incorporation at 1% and 4% levels in concrete mixtures containing a high volume of fly ash (40% and 60%) as a partial replacement for cement. The findings indicated that the addition of 1% nano-CaCO₃ significantly improved both the mechanical strength and durability of the concrete [33].

The present study aims to isolate and screen bacterial strains capable of producing a high yield of precipitated calcium carbonate (CaCO₃). The most potent isolate was selected for detailed morphological and molecular identification. Additionally, the study seeks to determine the optimal conditions for maximizing CaCO₃ precipitation. Microstructural analysis using scanning electron microscopy with energy-dispersive X-ray spectroscopy (SEM/EDX), X-ray diffraction (XRD), Fourier-transform infrared spectroscopy (FT-IR), transmission electron microscopy (TEM), and the N₂ desorption/adsorption isotherm (BET method) confirmed the presence of CaCO₃. Calcium carbonate nanoparticles (CaCO₃ NPs) were incorporated into the fabricated cement pastes to enhance and modify their cementitious properties beyond conventional performance. The prepared pastes were modified with varying dosages of CaCO₃ NPs at 0.0, 0.5%, 1%, and 1.5% by mass. To investigate the microstructural characteristics and phase composition of the modified pastes, selected specimens were analyzed using Scanning Electron Microscopy (SEM), X-ray Diffraction (XRD), and Differential Thermogravimetric Analysis (TGA/DTG).

## Materials and methods

### Isolation and cultivation of CaCO_3_-producing bacteria

A total of six soil samples were collected from various locations along the edges of Wadi El-Natron Lake in the Western Desert of Egypt in November 2023. A total of approximately 500 g of soil was collected from each sampling location at a depth ranging from 10 to 15 centimeters. The samples were cleaned by removing large stones and pebbles and then transported to the laboratory in sterile containers. To preserve bacterial viability, the samples were stored at 4 °C until further studies for bacterial isolation.

Ten grams of each soil sample was inoculated into 100 mL of B4 broth medium, which contained 4 g of yeast extract, 5 g of dextrose, 2.5 g of calcium acetate, and 1000 mL of distilled water. For solid media preparation, 1.5–2% (w/v) agar was added [34]. The pH of the medium was attuned to 8.2 using 1 N NaOH before sterilization. To prevent thermal decomposition, calcium acetate (Ca(CH_3_COO)_2)_ was sterilized separately through filtration using a sterile syringe filter and subsequently added to the medium. The prepared medium was then autoclaved at 121 °C for 20 min to ensure sterility.

The soil suspensions were incubated on a rotary shaker at 30 °C and 150 rpm for 7 days. After incubation, serial dilutions were performed ranging from 10⁻¹ to 10⁻⁶. The last three dilutions were selected for the spread plating technique on B4 agar plates. Colonies that formed white deposits indicative of calcium carbonate precipitation were sub-cultured onto fresh media to obtain pure cultures. Each colony was subsequently sub-cultured onto a fresh B4 agar plate to ensure the isolation of pure bacterial strains.

### Screening of purified isolates for high-yield of CaCO_3_

The isolated bacteria were inoculated into 100 mL of B4 broth medium, with the initial pH adjusted to 7.0. The flasks were incubated on a shaker at 30 °C and 150 rpm for seven days. The ultimate pH was assessed at the final stage of the incubation time.

After 7 days, the precipitates generated in the bacterial cultures were collected by centrifugation at 8000 rpm for 10 min at 4 °C. The precipitated calcium carbonate (CaCO₃) was carefully rinsed twice with sterilized distilled water and then dried in a hot air oven at 105 °C until a stable weight was attained. The dry weight of the precipitate was calculated [34]. Bacterial strains that exhibited a high yield of CaCO₃ precipitation were selected for further analysis.

### Morphological and biochemical bacterial identification

The bacterial isolate with the highest yield of CaCO₃ precipitation was further examined and identified. The isolate was stained and examined microscopically utilizing the Gram staining technique, as outlined by Wu et al. [19]. Catalase production was assessed following the procedure described by Loewen et al. [35].

Additionally, a urease activity test was performed by inoculating the bacterial isolate into lysogeny agar medium containing 10 g/L tryptone, 10 g/L NaCl, 5 g/L yeast extract, and 1.5-2% (w/v) agar. The growth medium was supplemented with 0.012 g/L phenol red indicator and 20 g/L urea, with the pH adjusted to 6.8–7.0. The agar plates were incubated at 37 °C for 24–48 h to assess urease activity [36].

To confirm sporulation, the bacteria were grown in a nutrient medium at 30 °C and 150 rpm for 24–48 h. Afterward, the bacterial culture was subjected to a water bath at 80 °C for 1 h to kill any vegetative cells, followed by re-culturing on a nutrient agar plate and re-incubation for an additional 24 h to confirm spore formation [37]. The morphological features of these spores were visualized using SEM and TEM.

The bacterial suspension from an overnight culture plate served as the inoculum for sporulation. It was evenly spread onto nutrient broth supplemented with 0.05 g/L MnSO₄·4 H₂O to promote sporulation [38]. Sporulating cells underwent heat treatment at 80 °C in a water bath for 20 min over several days to achieve complete cell lysis [37]. A uniform sample preparation protocol was applied for both Scanning Electron Microscopy (SEM) and Transmission Electron Microscopy (TEM). Cells were harvested by centrifugation at 2000×g for 10 min at 4 °C, followed by two washes with normal saline, each lasting 15 min. The specimens were then dehydrated using a graded ethanol series (30%, 50%, 70%, 90%, and 100%). For SEM analysis, samples underwent an additional wash with isoamyl acetate for 2 h [39] and were examined using a JEOL JSM-700 SEM (Japan) at the Central Laboratory, Faculty of Science, Benha University. For TEM analysis, dehydrated specimens were transferred to absolute acetone, embedded in Spurr’s resin, and incubated at room temperature for 4 h before polymerization in an oven at 65 °C for 24 h. Ultrathin sections were prepared using a Leica EM UC7 ultramicrotome and stained with uranyl acetate and alkaline lead citrate for 5–10 min [39], and visualized using a JEOL JMS-1200EX transmission electron microscope at the Faculty of Science, Ain Shams University.

### Molecular identification of selected bacterial isolates

The genomic DNA of bacterial isolate code D16 was extracted from cells cultured in LB broth and purified using the Gspin™ Total Extraction Kit, following the manufacturer’s instructions for bacterial DNA extraction. The 16 S rRNA gene of the isolate was subsequently amplified using a Thermocycler (Biometra Thermocycler, Germany) with universal primers 27 F (5′-AGA GTT TGA TCM TGG CTC AG-3′) and 1492R (5′-TAC GGY TAC CTT GTT ACG ACT T-3′), as determined by [40]. The amplified 16 S rRNA PCR product was subsequently sequenced with an Automated ABI 3730xl sequencer at Macrogen Inc., South Korea.

The PCR amplification was conducted under these parameters: an initial denaturation at 95 °C for 5 min, succeeded by 30 cycles comprising denaturation at 95 °C for 30 s, annealing at 55 °C for 2 min, and extension at 68 °C for 1.5 min. A concluding extension phase was performed at 68 °C for 10 min. The resultant PCR product was purified with the Montage PCR Clean-up Kit (Millipore).

The Big Dye Terminator Cycle Sequencing Kit v.3.1 (Applied Biosystems, USA) was employed for sequencing. The sequencing reaction was conducted at Macrogen Inc., Seoul, South Korea, and the sequencing product was analyzed using an Applied Biosystems 3730XL automated DNA sequencing system (Applied Biosystems, USA).

A sequence similarity search was conducted using the BLAST tool (http://www.ncbi.nlm.nih.gov/blast/) to compare the obtained 16 S rRNA sequence with existing sequences in the database. The highest aligned sequence from the BLAST search was used to identify the bacterial isolate.

### Optimization of different factors for high yield of CaCO_3_ precipitation

The optimal growth conditions for the bacterial isolate were determined to evaluate the mineralization yield. Various environmental parameters, including temperature, pH, calcium ion concentration, and urea concentration, were assessed to identify the conditions that maximize bacterial growth and calcium carbonate (CaCO₃) precipitation.

#### Effect of different pH

The target bacterial isolate was inoculated into 100 mL of a modified Urea-CaCl₂ broth medium containing 10 g of yeast extract, 25 mM CaCl₂, 5 g of NaCl, 20 g urea, and 1000 mL tap water per liter [41]. Before sterilization, the original pH of the medium was modified to 6, 7, 8, 9, 10, and 11 using 1 N NaOH.

The sterilized and inoculated flasks were incubated at 30 °C for 7 days with continuous shaking at 150 rpm. Following incubation, the precipitated calcium carbonate (CaCO₃) was gathered by centrifugation at 8000 rpm for 10 min at 4 °C. The precipitate was then washed, and dried, and its dry weight was measured as previously described.

#### Effect of different concentrations of calcium chloride

Various concentrations of calcium chloride (0.01, 0.025, 0.05, 0.1, 0.5, and 1 mol/L) were incorporated into the modified Urea-CaCl₂ broth medium. The target bacterial cells were inoculated into the prepared media and incubated with continuous shaking at 150 rpm at 30 °C for 7 days. Following the incubation period, the precipitate formed in each bacterial culture flask was collected by centrifugation, washed, and dried, and its dry weight was measured as previously described.

#### Effect of different incubation periods

Bacterial cells were inoculated into the modified Urea-CaCl₂ medium and incubated at 30 °C with continuous shaking at 150 rpm for varying incubation periods ranging from 1 to 7 days. Following each incubation period, the precipitated calcium carbonate (CaCO₃) was collected, washed, and dried, and its dry weight was measured as previously described.

#### Effect of different temperature

The modified Urea-CaCl₂ medium was inoculated with the bacterial culture and incubated at 10 °C, 20 °C, 30 °C, and 40 °C for 7 days with continuous shaking at 150 rpm. Following incubation, the precipitated (CaCO₃) was harvested, washed, and dried, and its dry weight was measured as previously described.

### Determination of specific growth rate

The modified Urea-CaCl₂ medium was inoculated with 1% (v/v) of standard inoculum containing 2.19 × 10^9^ viable cells/ml. The flasks were incubated for 7 days on a rotary shaker at 30 °C and 150 rpm. Specific growth rate (µ) (h^− 1^) and doubling time (td) (h) were calculated using the following formulas according to Doelle (1975). The following formulas were used to calculate these parameters: specific growth rate (µ) (h^− 1^) = (ln X –ln X0) (t - t0)^−1^, doubling time (td) (h) = ln2 (µ)^−1^ [42].

### Characterization of precipitated CaCO_3_

Transmission electron microscopy (TEM-2100, USA) operating at an accelerating voltage of 25 kV and scanning electron microscopy (SEM, Jeol JMS-700 EDX) at 10 kV were utilized to analyze the shape, morphology, and size of the precipitated calcium carbonate (CaCO₃).

Before imaging, the samples were diluted with the same volume of distilled water and subjected to sonication for 45 min using an ultrasonic probe instrument (Elma, Singen, Germany). Drops of the prepared suspension were then placed onto a coated copper grid and air-dried before TEM imaging. SEM was equipped with an energy-dispersive X-ray (EDX) analyzer for elemental composition analysis.

X-ray powder diffraction (XRD) analysis was conducted using a Philips 1830/40 X-ray diffractometer on finely powdered samples. The measurements were performed using Cu-Kα radiation (40 kV, 30 mA) with a Ni filter at a scanning speed of 0.005° 2θ s⁻¹. A time constant of 2 s was applied, and diffraction spectra were recorded over a 2θ range of 10°–60°. The crystalline phase and mineral composition of the precipitated CaCO₃ were determined through XRD analysis.

Fourier-transform infrared (FTIR) spectroscopy was performed using a Genesis-II FT-IR spectrometer with KBr-disc samples. Spectra were recorded over a wavenumber range of 400–4000 cm⁻¹ at the Chemistry Department, Faculty of Science, Benha University.

The N₂ desorption/adsorption isotherm, employing the Brunauer-Emmett-Teller (BET) technique, was used to ascertain the specific surface area of the precipitated CaCO₃. Measurements were conducted using a surface area and pore size analyzer (NOVA touch LX2, model NT2LX-2, Quantachrome, USA).

### Preparation of specimen

#### Materials and mixture proportions

Ordinary Portland Cement (OPC) Type I (42.5 N), supplied by Beni-Suef Cement Company (Beni-Suef, Egypt), and nano-calcium carbonate (NC) were used in the preparation of all specimens in this study. The chemical composition of the starting OPC binder was determined using X-ray fluorescence spectroscopy (XRF, PW-1400), as shown in Table 1. For the XRF analysis, 10 g of OPC powder was dried for 24 h, mixed with 1 g of borax, and then pressed into tablet form using a lead ring under a pressure of 3–12 tons/cm³.

The control paste (NC0.0) consisted of pure OPC, while three nanocomposite pastes (NC0.5, NC1.0, and NC1.5) were prepared by partially replacing OPC with 0.0, 0.5%, 1%, and 1.5% by weight of NC, respectively. All pastes were mixed using a constant water-to-powder ratio (W/P) of 0.32. Detailed mixture proportions are presented in Table 2. To ensure uniform dispersion, NC nanoparticles were first suspended in the mixing water and subjected to ultrasonic homogenization using a Biosafer 1200-98 ultrasonic device (220 V/50 Hz, 750 W) for 15 min at room temperature. The resulting NC suspension was then combined with the dry OPC using an EL-E automatic mixer to produce a homogeneous and workable paste.

**Table 1:** The oxide chemical composition for OPC (mass%)

Oxides	Mass,%
SiO_2_	19.4
Al_2_O_3_	4.65
Fe_2_O_3_	3.82
CaO	64.11
MgO	1.85
SO_3_	3.16
K_2_O	0.27
Na_2_O	0.31
Cl^−^	0.0
L.O. I	2.43
L.O.I = loss of ignition.	

The fresh pastes were cast into 1-inch cubic stainless-steel molds and cured under controlled conditions at room temperature and 98 ± 1% relative humidity for the first 24 h. Following demolding, the hardened composites were submerged in tap water and continuously cured at room temperature (≈ 28 °C) until the specified time intervals (1, 3, 7, 28, and 90 days) were reached.

**Table 2:** The mix design for investigated composites

Mix	OPC	NC	W/*p*
NC0.0	100	0	32%
NC0.5	99.50	0.50	32%
NC1.0	99.00	1.00	32%
NC1.5	98.50	1.50	32%

#### Testing and characterization

Physico-mechanical properties of all composites were assessed via estimating pore structure parameters, chemically combined water (Wn%), and compressive strength (CS). The pore-structure parameters were assessed via the measurement of bulk density (BD), total porosity (TP) percentage, and chemically combined water (Wn%) according to Ref [43]. According to ASTM C109, the average compressive strength for three cubic hardened composites was measured at different time intervals (1, 3, 7, 28, and 90 days) using a Seidner Riedinger compression machine (300 kN capacity).

**Fig. 1:**
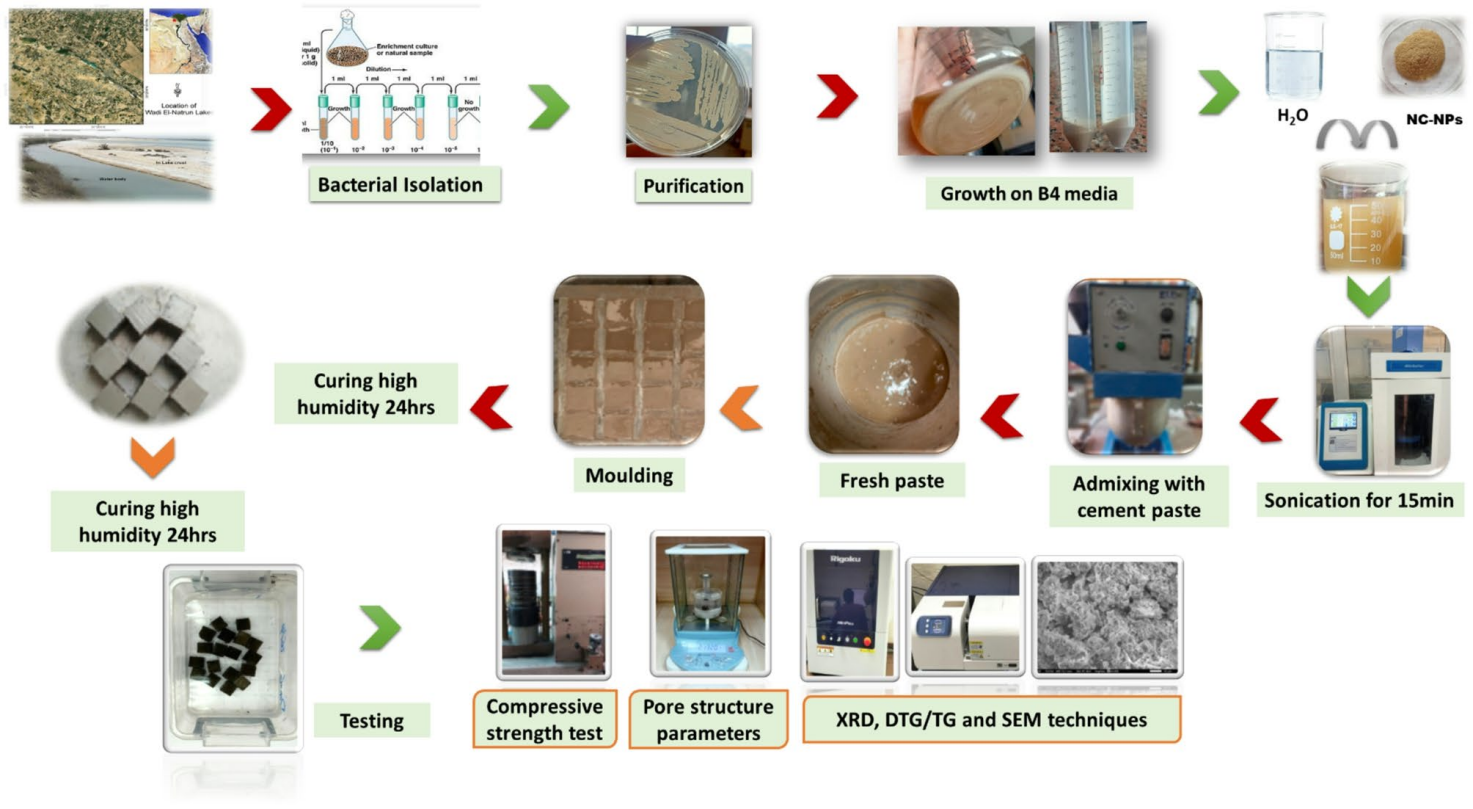
A graphic representation of the experimental program

Differential thermogravimetric analysis (DTG/TGA) techniques and X-ray diffraction (XRD) were utilized to determine the produced phases or unreacted phases for some selected composites (NC0.0 and NC0.5) cured at 1, 7, and 90-days. A scanning electron microscope (SEM) was used to investigate the morphology of the formed phases within the microstructure of these composites after curing at 1, 7, and 90-days. Figure 1 provides a summary of all the experimental procedures, including preparation of NC, admixing with cementitious blends, moulding, testing, and characterization. A graphic representation of the experimental study was shown in Fig. 1.

### Statistical analysis

Data were statistically determined using the IBM^®^ SPSS^®^ Statistics software version 21 on the premise of Duncan’s multiple range test at the 5% level. All analyses were performed in triplicate.

## Results and discussion

### Isolation and screening for CaCO_3_-producing bacteria

The results presented in Table 3 indicate that a total of twenty-four bacterial isolates were obtained from the six soil samples. Among these, isolate code D16 significantly increased than other isolates and exhibited the highest CaCO_3_ precipitation, yielding 0.404 g/100 mL, accompanied by high bacterial growth and a final pH of 9.09. This was followed by isolate code D2, which produced 0.356 g/100 mL of CaCO₃. In contrast, the lowest precipitation was recorded for isolate code D23, which yielded only 0.004 g/100 mL, with a final pH of 6.25 and minimal bacterial growth. Consequently, isolate code D16 was selected for further investigation due to its superior CaCO₃ precipitation when cultured in a B4 broth medium. These findings align with those reported by Ekprasert et al. [34], who observed that the CaCO₃ precipitated in all bacterial culture flasks exceeded 0.1 g/L, whereas the non-inoculated controls exhibited precipitation of less than 0.02 g/L. This confirms that CaCO₃ formation resulted from microbial activity rather than purely chemical reactions.

**Table 3:** Amount of precipitated CaCO_3_ using B4 broth media

Isolate code		Soil Sample	Final pH	Absorbance ($\:\varvec{\lambda\:}$=600 nm)	CaCO_3_ (g/100 ml)	Isolate code	Soil Sample	Final pH	Absorbance ($\:\varvec{\lambda\:}$=600 nm)	CaCO_3_ (g/100 ml)
D1		1st soil sample	8.02	+*	0.059^ghi**^ ± 0.007	D13	4th soil sample	8.92	+	0.153^de^ ± 0.009
D2			9.03	+++	0.356^b^ ± 0.02	D14		9.01	+	0.073^ghi^ ± 0.012
D3			9.02	+++	0.316^c^ ± 0.012	D15		8.92	+	0.159^d^ ± 0.02
D4			8.55	+ +	0.193^d^ ± 0.004	**D16**	5th soil sample	**9.09**	**+++**	0.404^a^ ± 0.026
D5			8.86	+	0.120^ef^ ± 0.021	D17		8.91	+++	0.286^c^ ± 0.065
D6		2nd soil sample	8.86	++	0.173^d^ ± 0.016	D18		8.76	+	0.075^ghi^ ± 0.004
D7			9.09	+	0.036^ijk^ ± 0.004	D19		8.88	+	0.070^ghi^ ± 0.008
D8			9.02	+	0.075^ghi^ ± 0.004	D20		8.85	+	0.079^gh^ ± 0.006
D9		3rd soil sample	8.06	+	0.076^ghi^ ± 0.004	D21	6th soil sample	6.54	+	0.015^jk^ ± 0.001
D10			9.00	+	0.046^hij^ ± 0.012	D22		7.20	+	0.044^hij^ ± 0.003
D11			9.02	+	0.1^fg^ ± 0.008	D23		6.25	+	0.004^k^ ± 0.001
D12			9.03	++	0.290^c^ ± 0.008	D24		8.91	++	0.172^d^ ± 0.002

* + low, ++ moderate, +++ high growth.

^**a, b^ values in the above column with the same letter do not differ significantly according to Duncan’s test at the 5% level. Mean ± standard division.

### Morphological and biochemical characterization of bacterial isolate

**Fig. 2:**
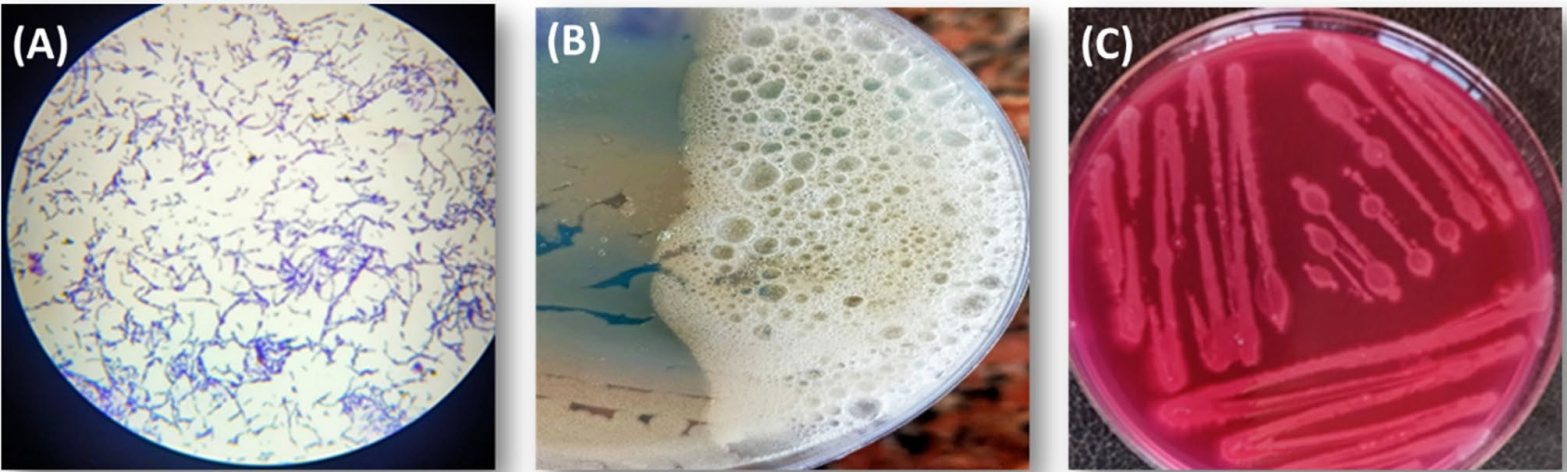
Morphological and Biochemical characterization; Gram stain (A), Catalase activity (B), Urease activity (C)

The data presented in Fig. 2A illustrate the results of the Gram staining process. The microscopic examination revealed that the bacterial isolate is Gram-positive and rod-shaped, arranged in chains. These findings are consistent with the observations reported by Stocks-Fischer et al. [44]. Additionally, the bacterial isolate exhibited catalase-positive activity, as shown in Fig. 2B. The presence of enzyme-generated oxygen bubbles, resulting from the decomposition of hydrogen peroxide into water and oxygen gas, confirmed catalase activity. This observation aligns with the findings of previous studies, such as those by Stocks-Fischer et al. [44].

As shown in Fig. 2C, the results indicated that the lysogeny agar medium changed from yellow to pink, providing a qualitative indication of urease enzyme activity. The pH increase observed was a consequence of the microbial conversion of urea into ammonia, as described by Algaifi et al. [36]. The urease enzyme catalyzes the bacterial hydrolysis of urea (CO(NH₂)₂), leading to a rise in pH, which promotes the production of carbonate ions (CO₃²⁻). In the presence of calcium ions, this process ultimately results in the precipitation of calcium carbonate (CaCO₃), contributing to the formation of mineralized calcium carbonate, as previously noted by Stocks-Fischer et al. [44].

Our experimental results also indicated that the D16 isolate is a spore-forming bacterium. In this context, Danish et al. demonstrated that under high pH conditions, up to pH 11, an actively growing bacterial cell, known as a vegetative cell, can transform into a spore [45]. When environmental conditions become favorable, such as a decrease in pH and the availability of nutrients, these bacterial spores can revert to their vegetative form within a few minutes [46].

**Fig. 3:**
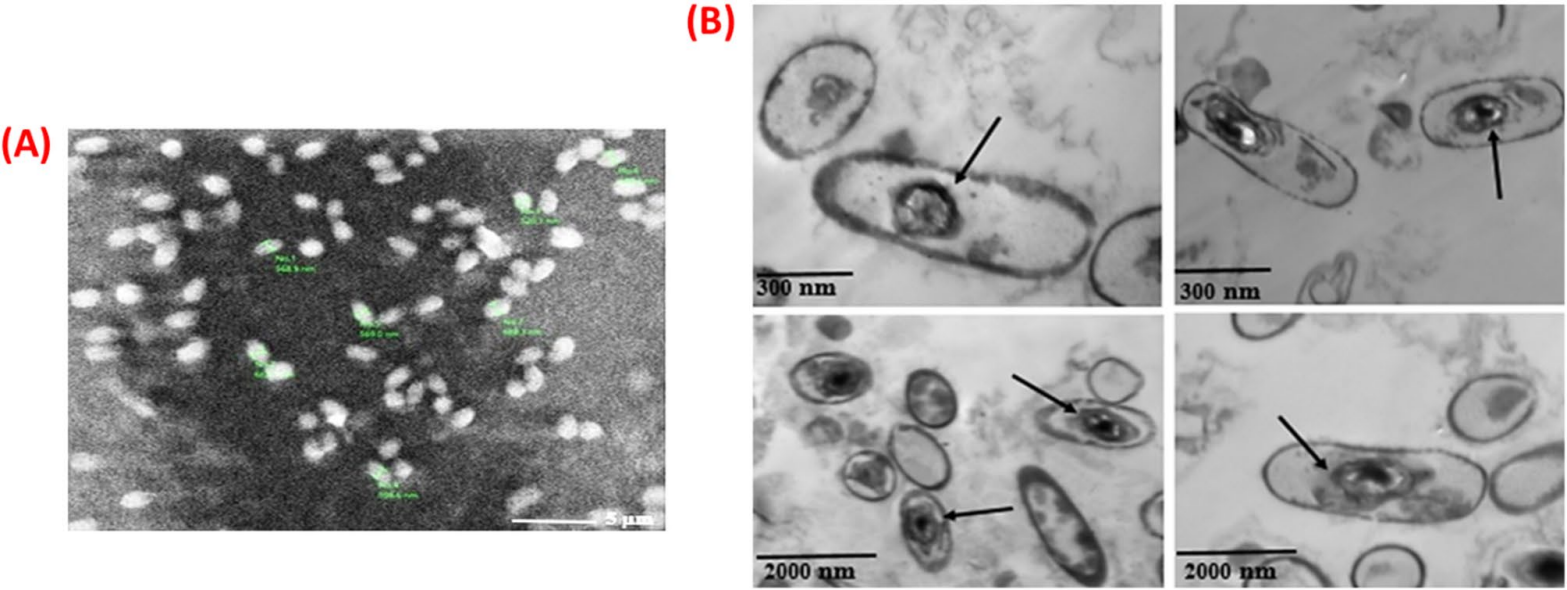
(A) SEM and (B) TEM images of *Bacillus tropicus* spores

The SEM image (magnification: ×3000) illustrates the morphological characteristics of *Bacillus tropicus* spores (Fig. 3A). The spores exhibit an oval to slightly elongated shape with relatively smooth surfaces. Size measurements indicate that the spore diameters range from approximately 568.9 to 685.3 nm, consistent with previously reported dimensions for *Bacillus* species, which range from 1.07 to 1.74 μm in length and 0.48 to 0.98 μm in diameter [47]. The observed successful sporulation supports the potential application of this strain in microbial-induced calcium carbonate precipitation (MICP) for crack sealing and enhancing concrete durability.

The TEM image (Fig. 3B) displays rod-shaped *Bacillus* cells with distinct electron-dense structures visible within the cytoplasm. These internal features are characteristic of endospores, recognized by their oval shape and multi-layered protective coats, which enhance resistance to heat and chemical stresses. The presence of these endospores confirms the bacterial transition into a dormant phase, indicative of their ability to survive environmental fluctuations and underscoring their adaptability and robustness [48].

### Molecular identification of selected bacterial isolate

**Fig. 4:**
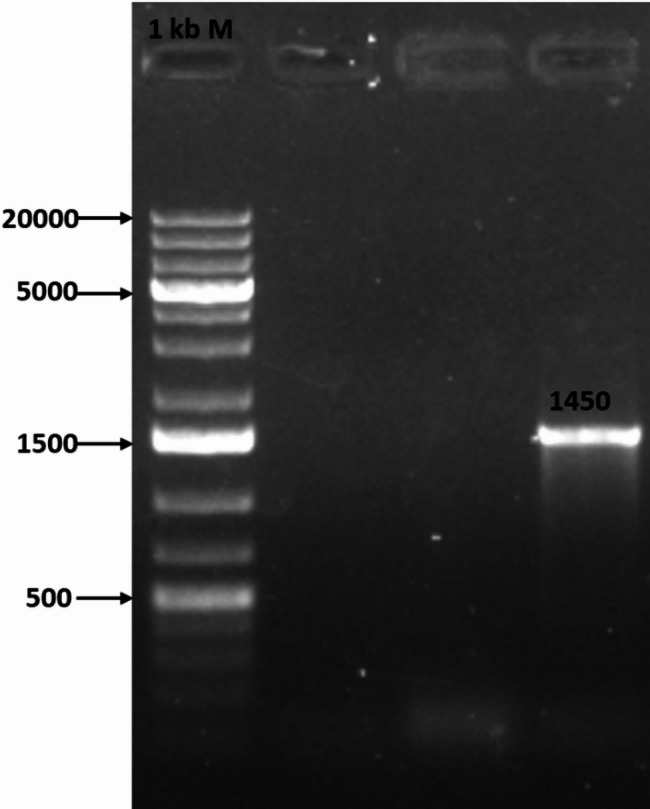
Agarose gel 1% showing the PCR product for 16 S gene; (M: 1kbp DNA ladder)

The obtained fragment size that was separated on 1% agarose gel electrophoresis was determined according to the DNA ladder. A fragment of 1450 bp for the isolate is observed in Fig. 4.

**Fig. 5:**
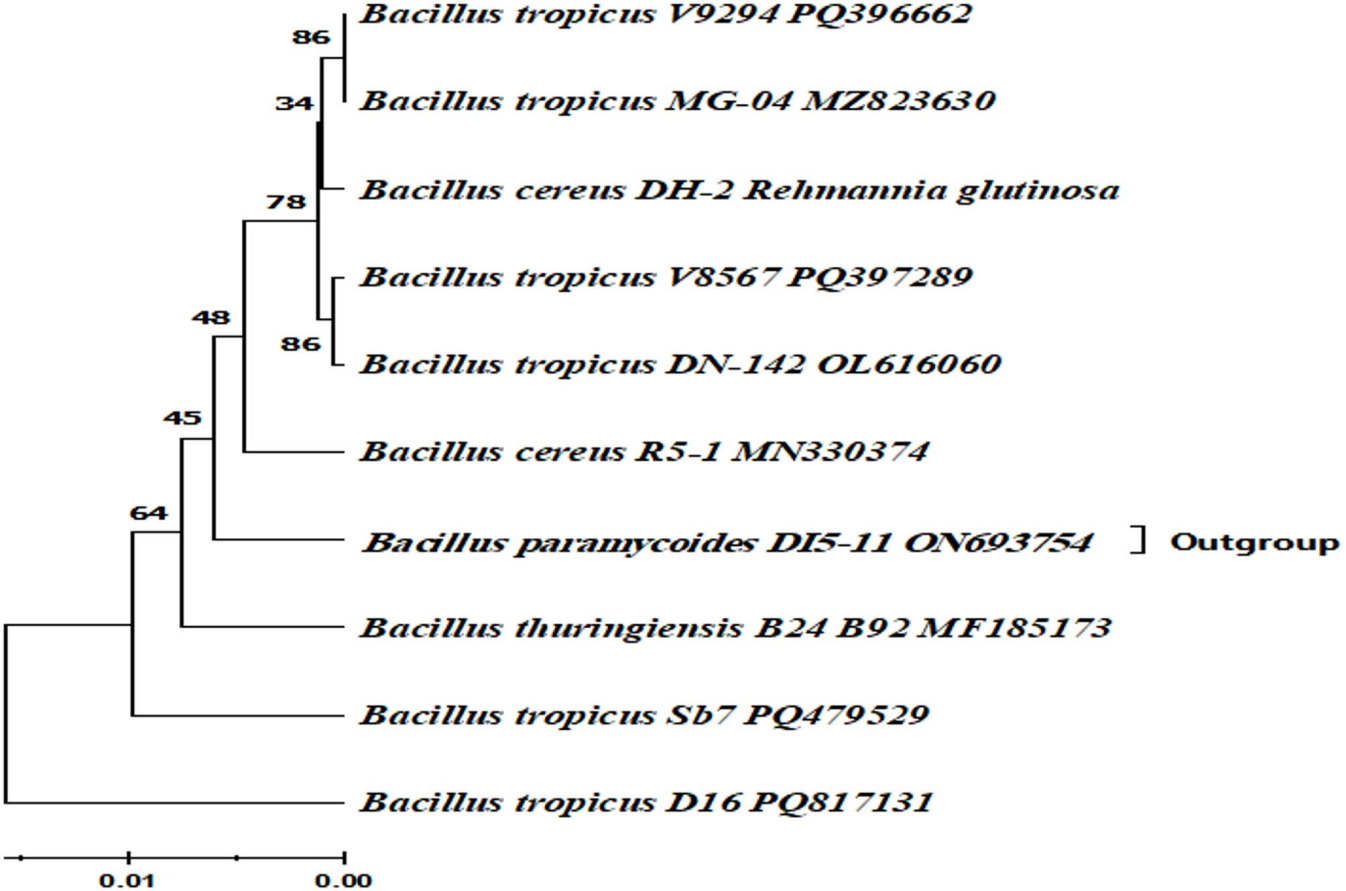
Phylogenetic tree constructed using 16 S rRNA sequence of D16 isolates using the maximum composite likelihood method (Evolutionary analyses were conducted using MEGA11)

Figure 5 illustrates the phylogenetic tree, which highlights the position of the identified strain to other strains. Molecular analysis based on 16 S rRNA gene sequencing confirmed that the most effective isolate is *Bacillus tropicus* strain D16. This strain has been deposited in the GenBank database under the accession number PQ817131.

### Optimization of different factors for high yield of CaCO_3_ precipitation

#### 3.4.1. Effects of different pH

**Fig. 6:**
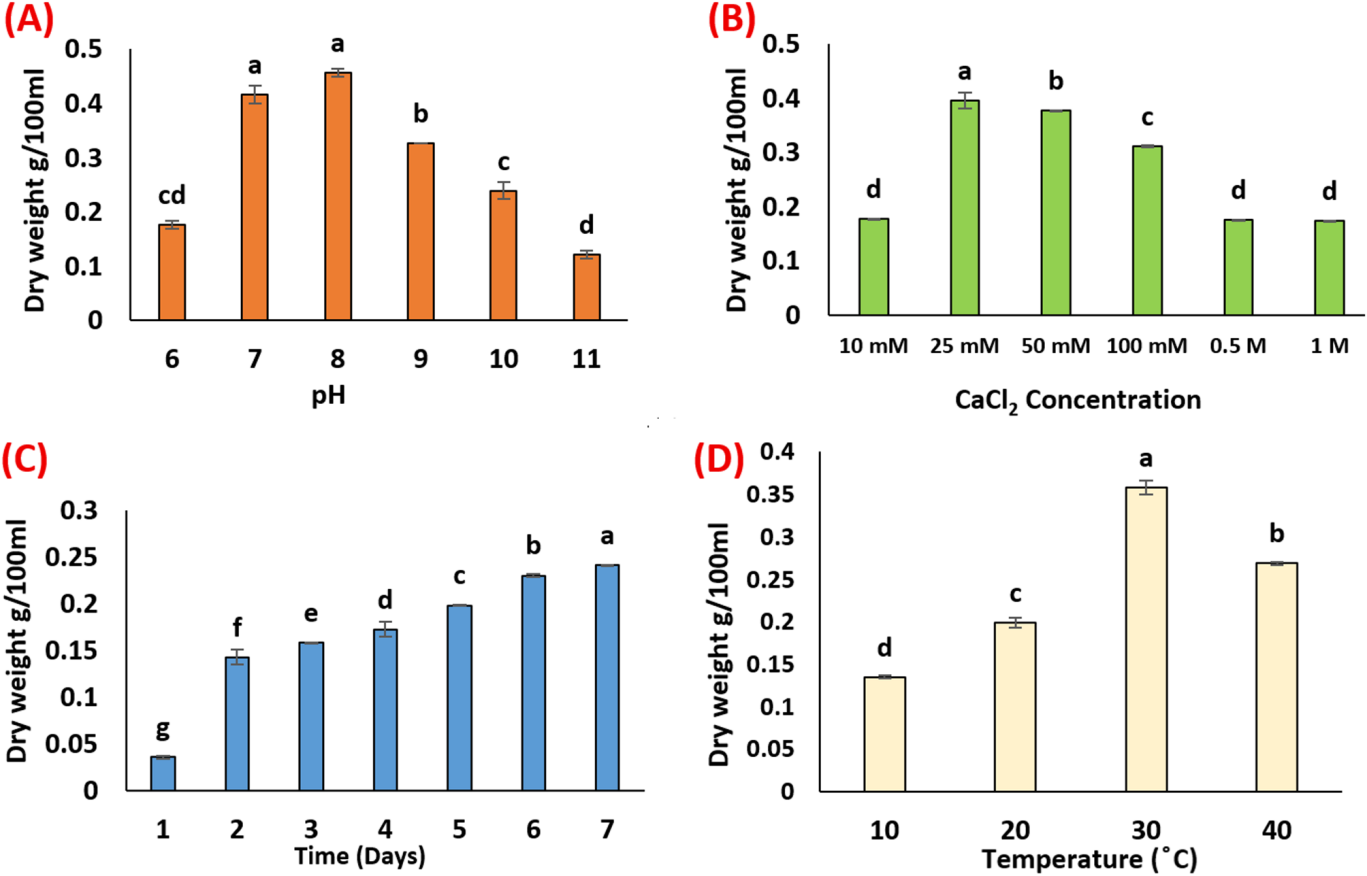
The factors affecting the amount of CaCO_3_ precipitation: pH (A); different concentrations of CaCl_2_ (B); incubation period (C), and temperature (D); ^a, b^ values in the above column with the same letter do not differ significantly according to Duncan’s test at the 5% level. Bar indicated to ± standard division

The CaCO₃ precipitate was collected and weighed after a 7-day incubation period, with the results presented in Fig. 6A. The data revealed a general increase in the amount of precipitate as the pH values rose from 6 to 8. The highest yield of CaCO₃ (0.456 g/100 mL) was significantly observed at pH 8, followed by a decline in precipitation as the pH increased from 9 to 11, with values of 0.3257 g/100 mL and 0.1214 g/100 mL, respectively.

Environmental pH is an important regulator of survival, metabolic rate, and overall microbial processes in concrete and microbial-induced calcium carbonate precipitation (MICP) [19]. In the context of MICP, the precipitation of CaCO₃ is significantly impacted by the pH levels. Bacteria that produce the enzyme urease, known as ureolytic bacteria, increase the pH of the medium, leading to the formation of CO₃²⁻ ions. This occurs through the hydrolysis of urea (CO(NH₂)₂) into ammonia ions (NH₄⁺) [44]. A higher concentration of CO₃²⁻ ions facilitate the precipitation of CaCO₃. The lime-carbonic acid equilibrium, as described in the following equations, illustrates that CO₃²⁻ ions are in a dynamic equilibrium with hydrogen carbonate ions and carbonic acid.

$$\:\text{C}{\text{O}}_{2}+{\text{H}}_{2\:}\text{O}\:\:\leftrightarrow\:\:{\text{H}}^{+}+\:{\text{H}}_{2\:}{\text{C}\text{O}}_{3}\:\:\:\:\:\:\:\:\:\:\:\:\:\:\:\:\:\:\:\:\:\left(1\right)$$

2 $$\:{\text{H}}_{2}{\text{C}\text{O}}_{3}\:\leftrightarrow\:\:{\text{H}}^{+}+\text{H}\text{C}{\text{O}}_{3}^{-}$$

3 $$\:\text{H}\text{C}{\text{O}}_{3}^{-}\:\leftrightarrow\:\text{C}{\text{O}}_{3}^{-2}+\:{\text{H}}^{+}$$

4 $$\:{\text{C}\text{a}}^{+2\:}+\text{C}{\text{O}}_{3}^{-2\:\:}\leftrightarrow\:{\text{C}\text{a}\text{C}\text{O}}_{3}$$

Environmental pH has a key function in determining the stability of this equilibrium and influencing the ratios of the carbonic acid species [49]. Changes in pH can shift the balance between carbonate ions, bicarbonate ions, and carbonic acid, thereby affecting the efficiency of microbial-induced calcium carbonate precipitation (MICP) and the overall process of mineralization.

#### Effect of different concentrations of calcium chloride

The amount of precipitated CaCO₃ in this treatment was collected, weighed, and presented in Fig. 6B. The highest amount of calcite (0.396 g/100 mL) was significantly observed at a CaCl₂ concentration of 25 mM, followed by a decline in precipitation as the concentration increased up to 50 mM. The optimal CaCl₂ concentration was determined to be 25 mM. This can be attributed to the increased urease activity at this concentration, which enhances the absorption of calcium ions onto the surface of bacterial cells. This occurs due to the electrostatic interaction between positively charged calcium ions and negatively charged bacterial cell walls, resulting in the precipitation of larger amounts of CaCO₃. However, at concentrations exceeding 50 mM, urease activity is inhibited, leading to a reduction in CaCO₃ precipitation [50].

#### Effect of different incubation periods

The ability of microorganisms to produce CaCO₃ precipitation is influenced by different incubation periods, as shown in Fig. 6C. A white powder began to form in the medium immediately, and its intensity increased as the incubation period progressed. Based on the findings of our experiment, 7 days was identified as the optimal incubation period for the CaCO₃ precipitation process, resulting in a significant yield of 0.241 g/100 mL. The amount of precipitate generally showed an increasing trend with longer incubation times, ranging from 0.035 g/100 mL at 1 day to 0.241 g/100 mL at 7 days. In this context, Algaifi et al. observed that as the incubation period progresses, the concentration of bacterial cells increases, providing more nucleation sites for the precipitation of calcium carbonate [36]. According to this concept, a sufficient bacterial cell population is necessary to ensure effective self-healing. In the absence of bacterial cells, urea cannot be metabolized, thus preventing the formation of calcium carbonate. The ureolytic activity is highly influenced by the bacterial cell concentration, as it facilitates urea hydrolysis into carbonate and ammonia ions, which are crucial for the precipitation process.

#### Effect of different temperatures

The data presented in Fig. 6D show the dry weight of the precipitate at different temperatures (10, 20, 30, and 40 °C) in the modified Urea-CaCl_2_ media. The highest amount of precipitate (0.358 g/100 mL) was obtained at 30 °C, while a decrease in precipitate formation was observed at 10 °C (0.135 g/100 mL) and 40 °C (0.269 g/100 mL). These results indicate that the bacteria have a limited capacity to adapt to both high and low-temperature environments, as they exhibit optimal growth within a specific temperature range. Outside of this range, except spore-forming bacteria, most bacterial strains fail to thrive or even perish [51]. Temperature tolerance is a critical factor for bacterial survival in concrete, as elevated temperatures may inhibit bacterial cell growth [52].

### Bacterial growth rate

**Fig. 7:**
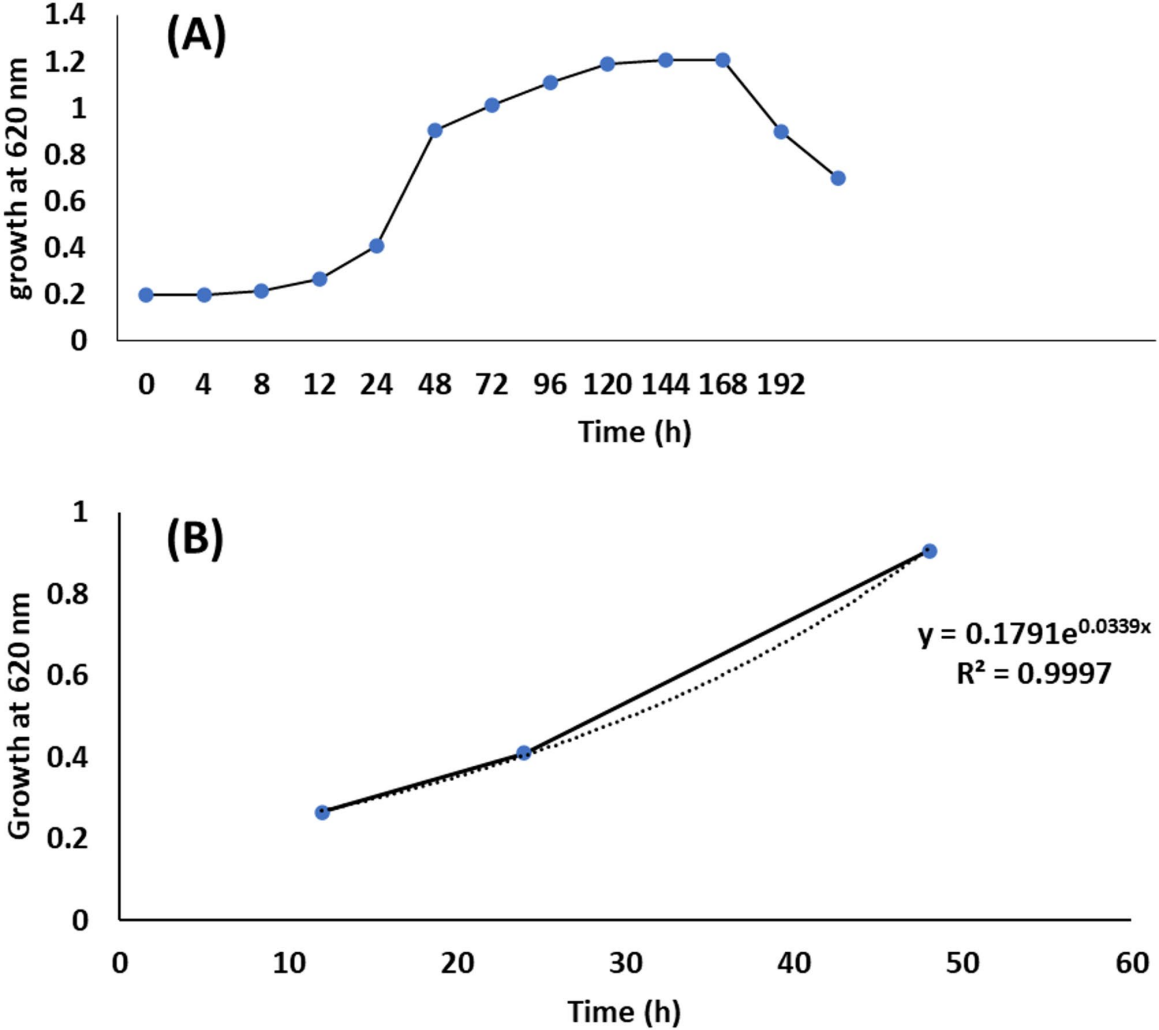
Growth pattern of *Bacillus tropicus* strain D16: Sigmoidal growth curves of *Bacillus tropicus* strain D16 (A); Correlation coefficient between the optical density of the growth and incubation time in log phase (B)

Results in Fig. 7 revealed that lag phase of *Bacillus tropicus* strain D16 was from 0 to 12 h of incubation period and grew exponentially during the first 12–48 h. The stationary phase was from 2 to 7 days, whereas the decline phase was after 7 days. The growth parameter was calculated at the log phase of growth curves. The specific growth rate (µ) and doubling times (td) were 0.0339 h^− 1^ and 20.44 h.

### Characterization of precipitated CaCO_3_

#### TEM and SEM/EDX characterization

**Fig. 8:**
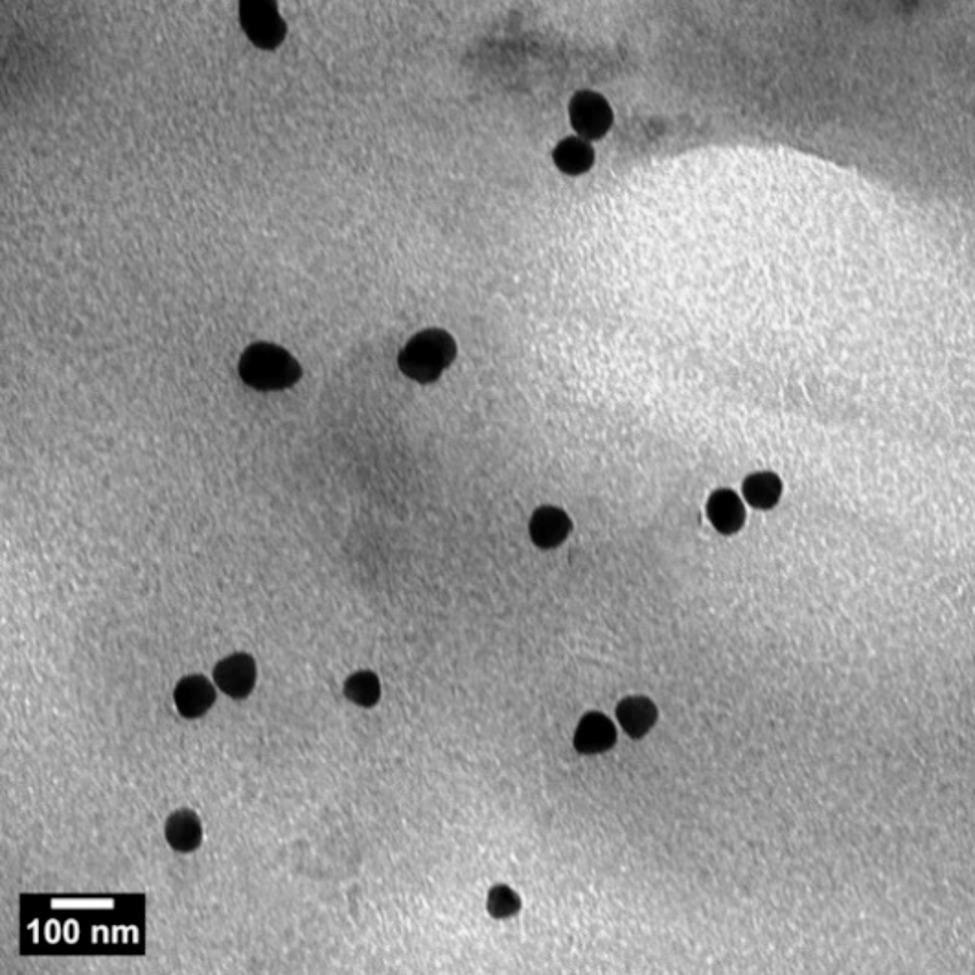
HR-TEM of CaCO_3_ nanoparticles produced by *Bacillus tropicus* strain D16

**Fig. 9:**
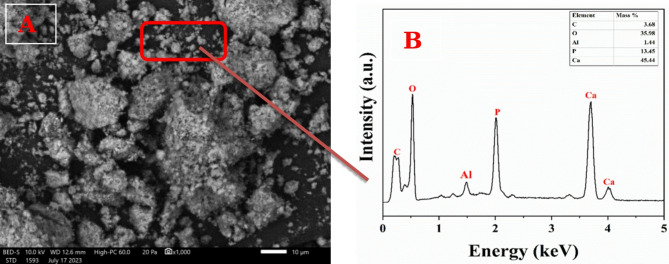
The morphology of precipitated CaCO_3_ nanoparticles produced by *Bacillus tropicus* strain D16; FE-SEM images (A); EDX patterns of CaCO_3_ (B)

The morphology and dispersion of precipitated CaCO_3_ nanoparticles were examined using high-resolution transmission electron microscopy (HR-TEM) (Fig. 8) and field emission scanning electron microscopy (FE-SEM) (Fig. 9). As shown in Fig. 8, the CaCO_3_ particles exhibit rhombohedral structures that aggregate to form spherical and semispherical shapes. The particle size of the CaCO_3_ particles, as observed from the TEM image, ranges from 50 to 65 nm.

Figure 9A presents FE-SEM micrographs of the CaCO_3_ nanoparticles, showing their spherical shape along with pronounced agglomerations.

According to Wei et al., five distinct calcium carbonate crystal morphologies were identified during microbial-induced calcium carbonate precipitation (MICP): cubic, rhombic, polygonal plate-like, spherical, and irregular forms. Among these, the cubic, rhombic, and polygonal morphologies were the most frequently induced by bacterial isolates, whereas the spherical and irregular types appeared less commonly. The study emphasized that the variation in crystal shape corresponds to differing physicochemical properties of the resulting mineral aggregates [53]. Similarly, Gu et al. observed a time-dependent transformation in calcium carbonate morphology during MICP. Initially, spherical or ellipsoidal particles formed around bacterial cells, indicating nucleation facilitated by bacterial surfaces. These early aggregates subsequently evolved into irregular lumps and, over time, transformed into well-defined rhombohedral crystals. By day 7, the dominant morphology was calcite, reflecting a structural transition from the metastable vaterite phase to the thermodynamically stable calcite form [54]. Frankel and Bazylinski further demonstrated that the mineral compositions resulting from biologically induced processes exhibit considerable heterogeneity, reflecting the diversity of formation environments. This variability encompasses external morphology, often poorly defined, along with differences in water content, trace and minor element compositions, crystal structure, and particle size [55]. Figure 9B illustrates the results of energy-dispersive X-ray (EDX) analysis performed at an accelerating voltage of 10 kV. The EDX spectrum indicates the chemical composition of the CaCO_3_ nanoparticles, with mass percentages of calcium (Ca), carbon (C), and oxygen (O) being 27.14%, 7.34%, and 53.84%, respectively. This analysis confirms that the precipitated CaCO_3_ nanoparticles are of high purity.

#### X-ray diffraction patterns (XRD)

**Fig. 10:**
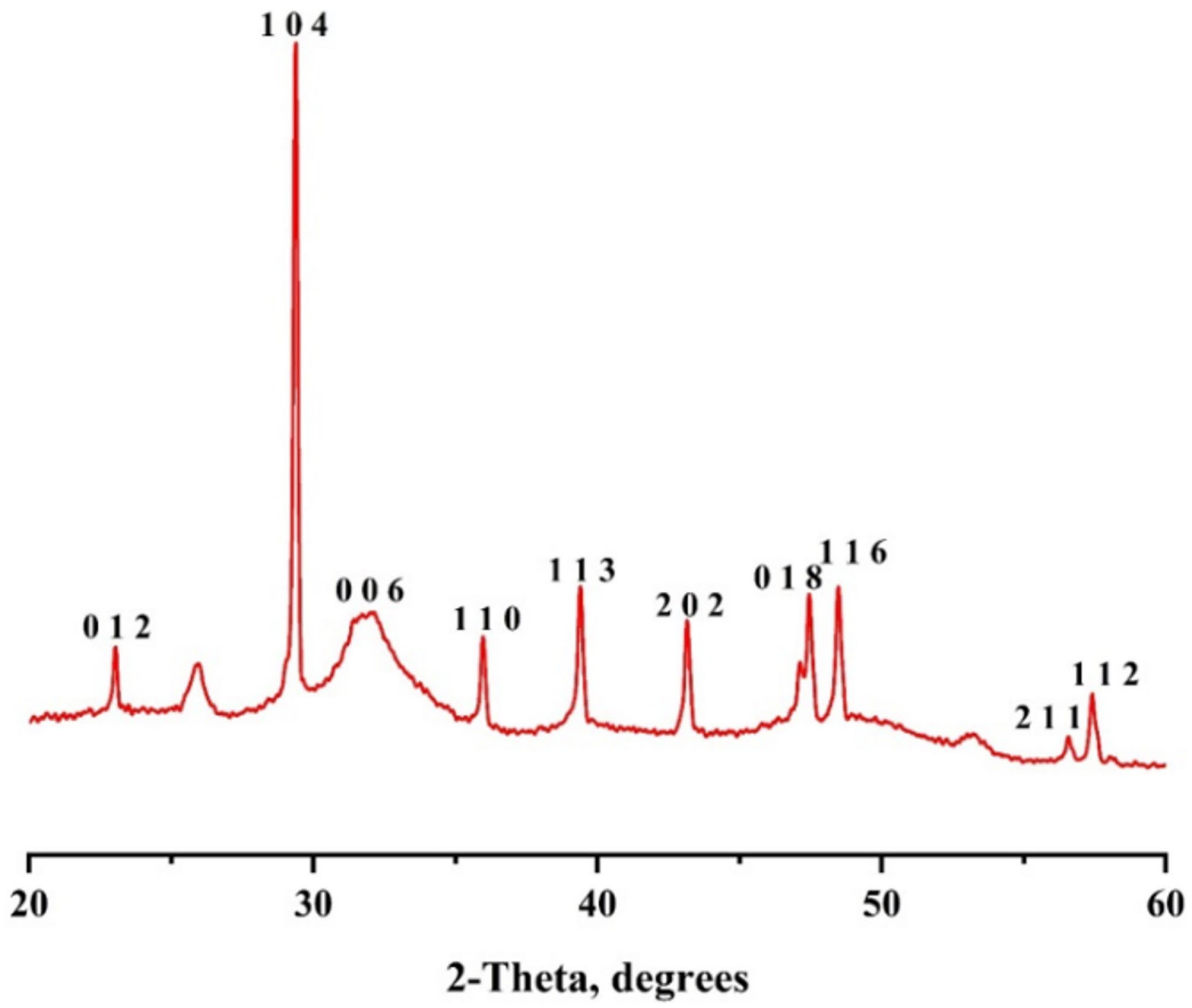
XRD patterns of CaCO_3_ produced by *Bacillus tropicus* strain D16

Figure 10 illustrates the X-ray diffraction (XRD) pattern of CaCO_3_ precipitated by the D16 bacterial isolate. The results indicate that the diffraction peaks of the crystalline nano-CaCO_3_ fall within the range of 20°–60°. Furthermore, the crystal structure of CaCO_3_ is identified as hexagonal, corresponding to the Joint Committee on Powder Diffraction Standards (JCPDS) card no. 21–00992. The estimated lattice parameters are a = 4.99365 nm, b = 4.99365 nm, and c = 17.09966 nm, with α = β = 90° and γ = 120°. The average particle size of the precipitated CaCO_3_ was determined to be 68.81 nm using the Debye-Scherrer formula.

#### Infrared (IR) spectral analysis

**Fig. 11:**
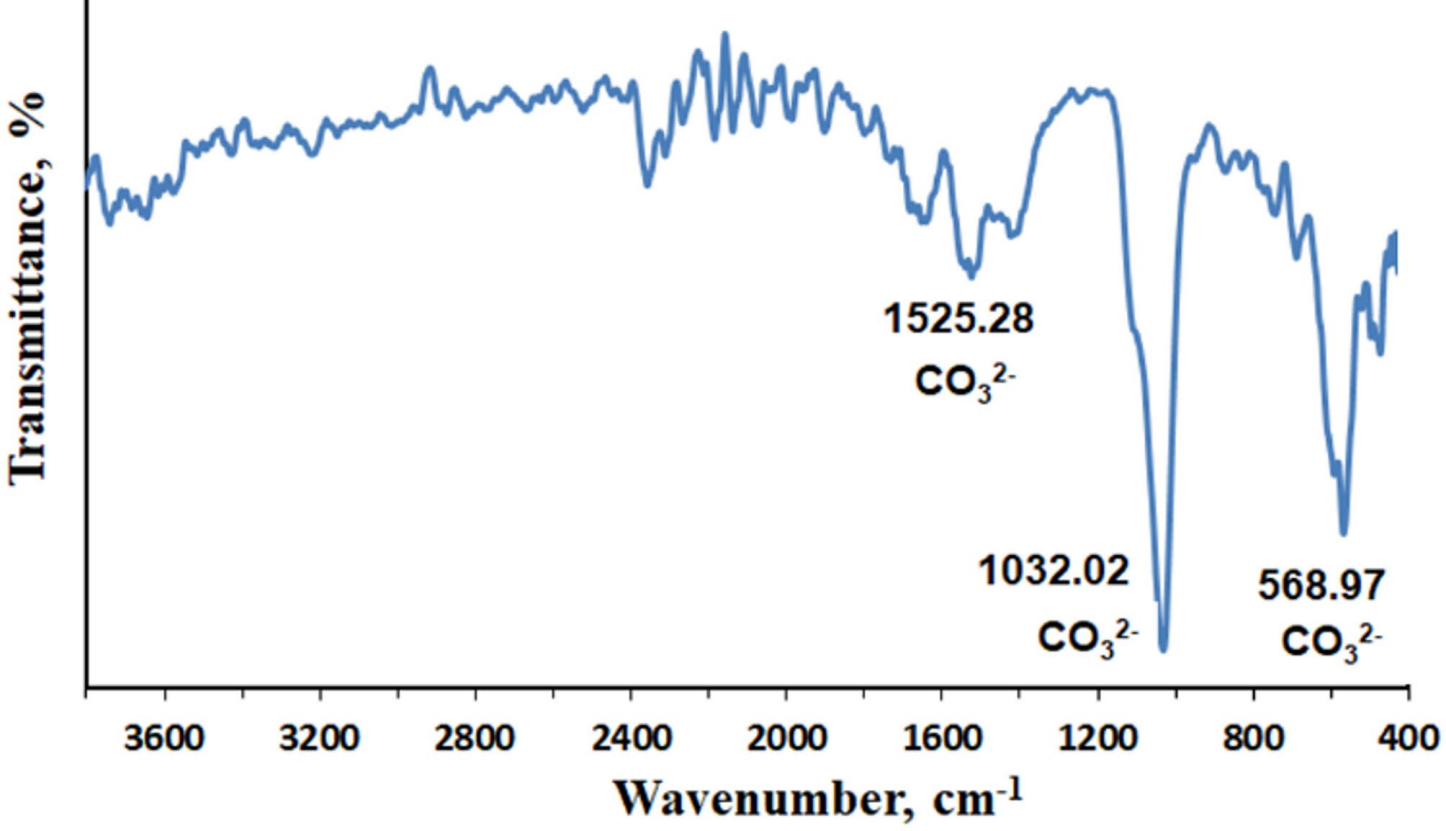
FT-IR analysis of CaCO_3_ produced by *Bacillus tropicus* strain D16

Figure 11 displays the FT-IR spectrum of precipitated CaCO_3_. There are three major peaks, which are 1525.28 cm^− 1^, 1032.02 cm^− 1^ and 568.97 cm^− 1^ respectively. The three peaks indicate the vibration of CO_3_^2−^ [56].

#### Surface area characterization

**Fig. 12:**
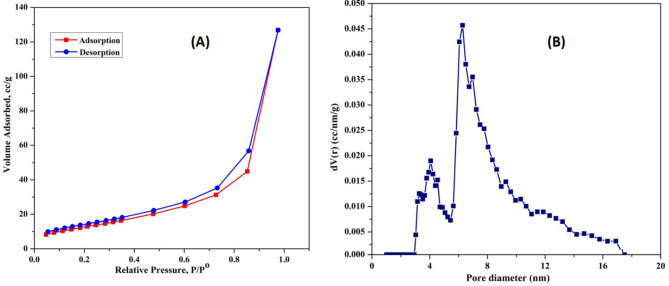
N_2_ adsorption/desorption of CaCO_3_ (A); and pore size distribution of CaCO_3_ produced by *Bacillus tropicus* strain D16

The N₂ adsorption/desorption isotherms and pore size distribution curves of the precipitated CaCO₃ are presented in Fig. 12. The N₂ adsorption/desorption isotherm exhibits a hysteresis loop at relative pressures (P/P^₀^) near unity, indicating the presence of extensive mesopores and macropores, which can be classified as type IV according to Brunauer’s categorization. The BET surface area, average pore size, and average pore volume of the CaCO₃ precipitate were determined to be 48.455 m²/g, 2.269 nm, and 0.1861 cc/g, respectively (Fig. 12A). The Barrett-Joyner-Halenda (BJH) pore size distribution curve, illustrated in Fig. 12B, exhibits two distinct peaks: the first peak at 4.2 nm and the second at 6.8 nm. These findings confirm that the precipitated CaCO₃ primarily consists of mesopores, following the classification criteria: micropores (0 nm < pore diameter < 2 nm), mesopores (2 nm < pore diameter < 50 nm), and macropores (pore diameter > 50 nm) [57].

### Characterization methods of cement composites containing nano-CaCO_3_ particles

#### Compressive strength (CS)

**Fig. 13:**
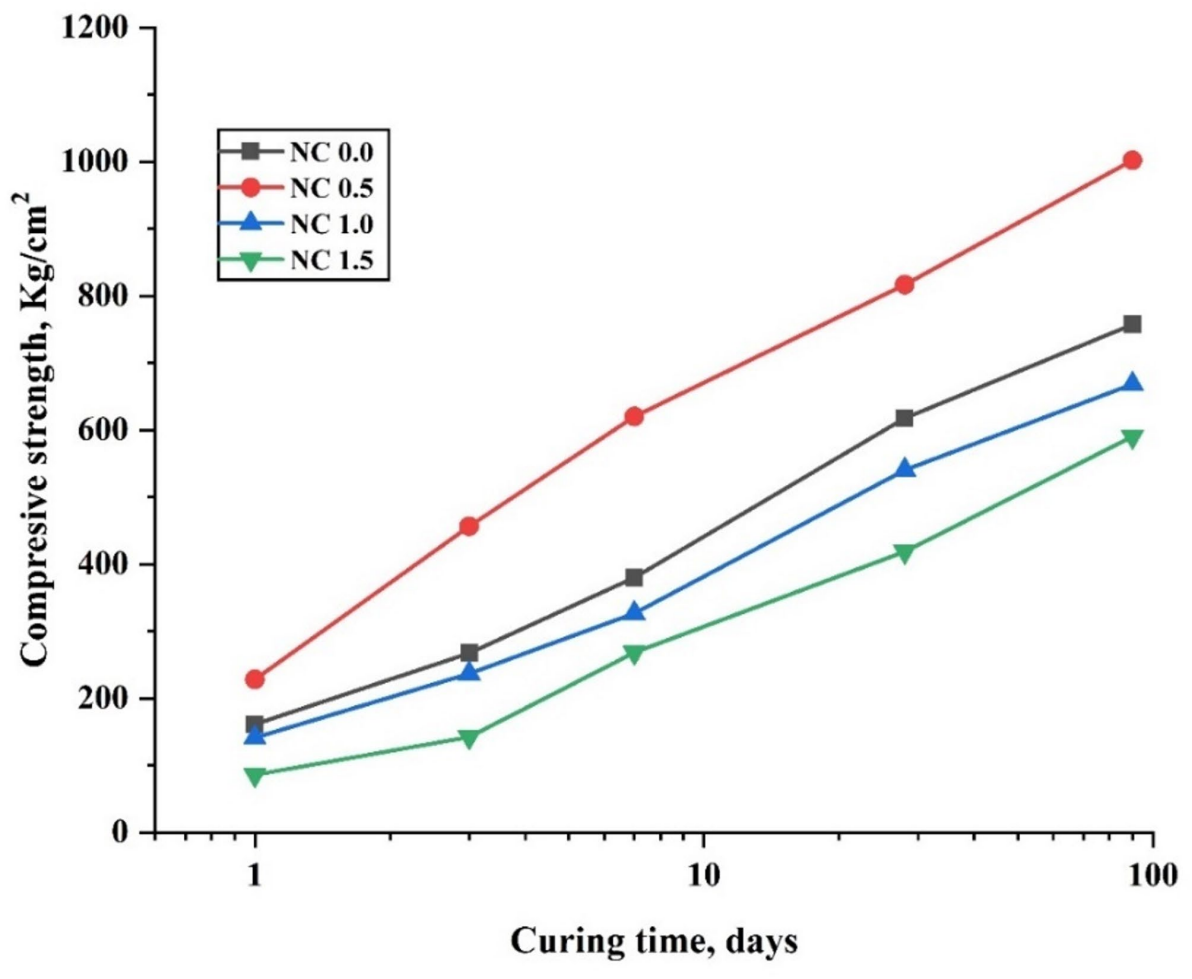
Compressive strength values at various ages of hydration for cement composites free from NC NPs and containing 0.5, 1.0, and 1.5% NC NPs

Figure 13 presents the graphical representation of compressive strength (CS) values for hardened composite pastes prepared with varying nano-CaCO₃ (NC) contents: NC0.0 (100% OPC), NC0.5 (99.5% OPC + 0.5% NC), NC1.0 (99% OPC + 1.0% NC), and NC1.5 (98.5% OPC + 1.5% NC), measured at different curing intervals (1, 3, 7, 28, and 90 days). As expected, the CS values of all composite pastes increased with curing time, primarily due to the progression of the hydration reaction and the consequent formation of binding phases such as calcium silicate hydrate (C–S–H), calcium aluminate hydrate (C–A–H), and calcium aluminosilicate hydrate (C–A–S–H). Notably, at all curing ages, the NC0.5 composite exhibited the highest CS values compared to the other mixtures (NC0.0, NC1.0, and NC1.5). The improved performance of NC0.5 is attributed to the consumption of calcium hydroxide (Ca(OH)₂) during hydration, particularly at early ages. Furthermore, the enhancement can be explained by the filler and nucleation effects of nano-CaCO₃. When added in small quantities and well-dispersed, these nanoparticles provide a high surface area that promotes the precipitation of hydration products, acting as nucleation sites and thereby accelerating the hydration process [58]. Specifically, the NC0.5 composite showed approximately 42%, 71%, 63%, 32%, and 33% higher CS values than the control (NC0.0) after 1, 3, 7, 28, and 90 days of curing, respectively. Conversely, higher NC contents (1.0% and 1.5%) led to a reduction in CS. This decline is attributed to the agglomeration of nanoparticles within the paste, driven by stronger van der Waals forces relative to cement particles, which hinders their uniform dispersion [59]. Additionally, calcium carbonate may react with tri-calcium aluminate (C₃A) to form monocarbonate, a compound characterized by strong hydrogen bonding between oxygen atoms and interlayer H₂O in CO₃²⁻ groups [60]. It may also influence the composition and stability of the AFm-phase [61]. These interactions, along with nanoparticle agglomeration, likely contribute to the diminished performance observed in composite pastes with higher NC content.

#### Pore structure parameters

**Fig. 14:**
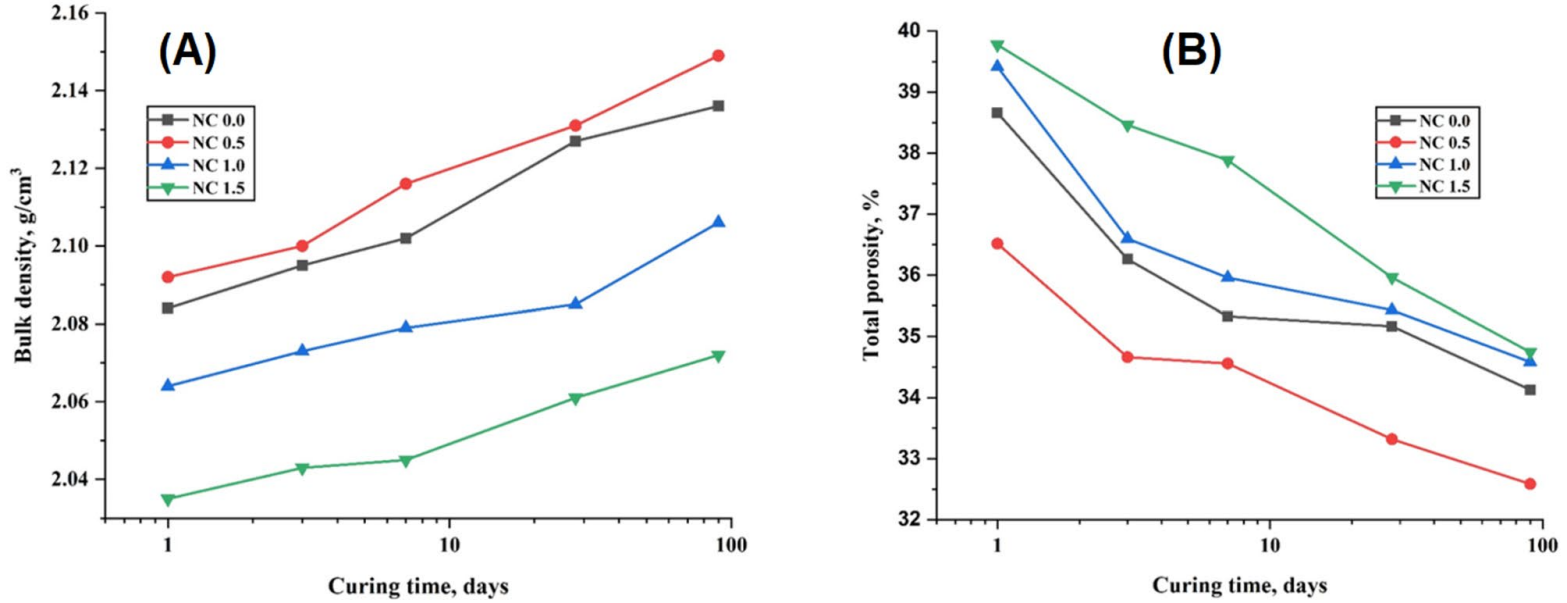
Bulk density (A); Total porosity (B) values at various ages of hydration for cement composites free from NC NPs and containing 0.5, 1.0, and 1.5% NC NPs

To elucidate the mechanical behavior of the prepared composite pastes, it is essential to assess their porous structure through measurements of bulk density (BD, g/cm³) and total porosity (TP, %). Figure 14A, B depicts the BD and TP values, respectively, for NC0.0, NC0.5, NC1.0, and NC1.5 composite pastes over a curing period of up to 90 days. As illustrated, increasing the NC content up to 0.5% led to a noticeable reduction in TP and a corresponding increase in BD. These findings align with the observed mechanical performance, indicating that the optimal NC dosage (0.5%) enhances the structural integrity of the composites. Specifically, the NC0.5 composite exhibited the lowest total porosity (4.54%, Fig. 14B) and the highest bulk density (an increase of 6.01%, Fig. 14A) compared to the NC0.0 control after 90 days of curing. This improvement in the porous structure is attributed to the accelerated cement hydration facilitated by NC, which resulted in the formation of abundant hydration products. These products effectively filled the pore spaces within the initially loose network structure surrounding the cement grains, thereby decreasing TP and increasing BD [62].

However, when the NC content exceeded 0.5%, a decline in compressive strength was observed, consistent with the porosity data. The increased NC content (in NC1.0 and NC1.5) may have inhibited the transformation of ettringite (AFt) into monosulfate (AFm), leading to internal stress due to excessive AFt formation. This stress could compromise the matrix integrity and contribute to the observed reduction in mechanical strength.

#### Hydration kinetics of composite pastes

**Fig. 15:**
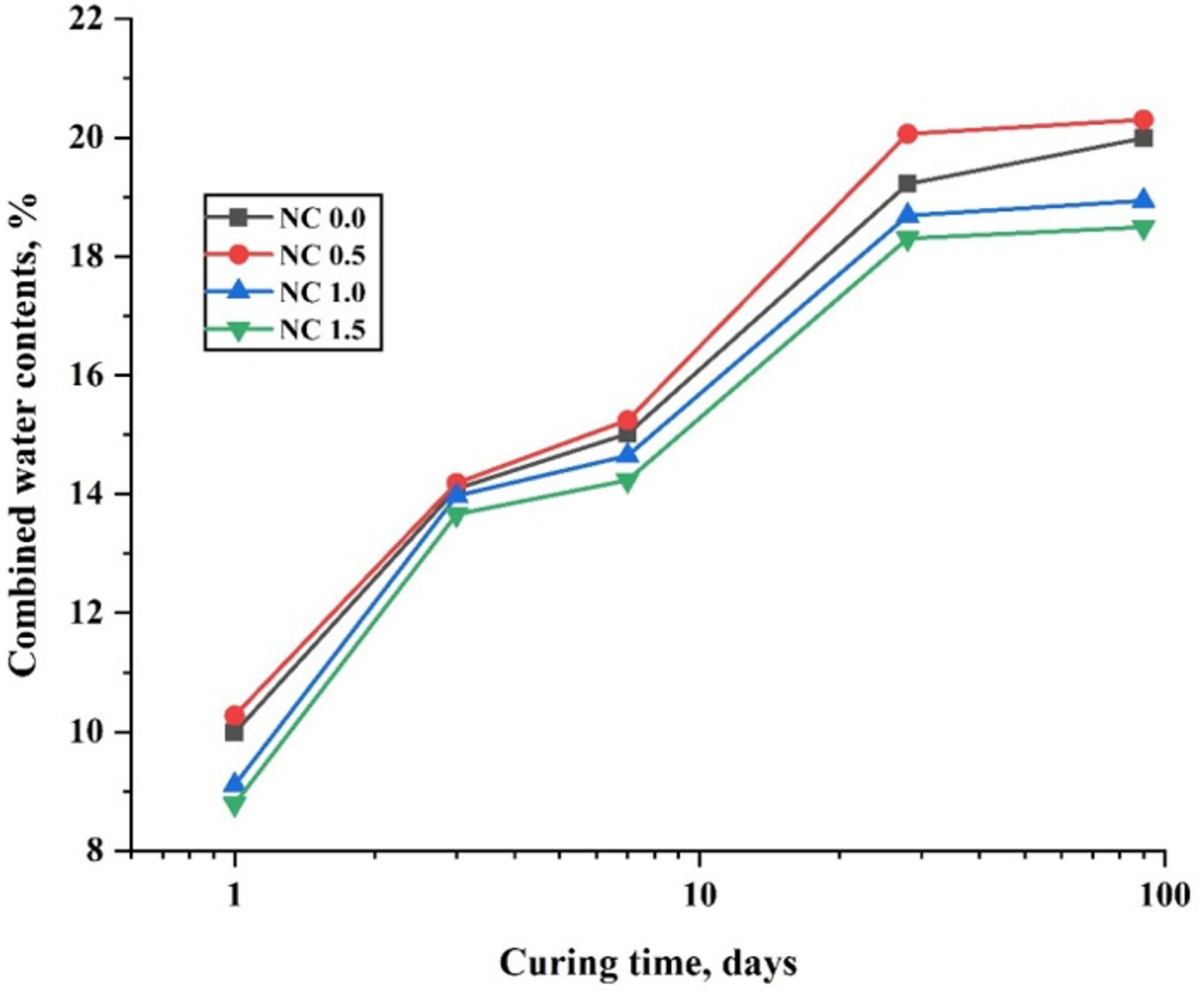
Chemically combined water% % at various ages of hydration for cement composites free from NC NPs and containing 0.5, 1.0, and 1.5% NC NPs

The hydration kinetics of the control paste (NC0.0) and nano-CaCO₃-modified composite pastes (NC0.5, NC1.0, and NC1.5) were assessed by determining the chemically combined water content (Wn, %). The results, presented in Fig. 15, illustrate the variation in Wn (%) for all pastes up to 90 days of hydration. The data indicate that the Wn content of the NC0.5 composite increased significantly by 2.88%, 1.10%, 1.55%, 4.39%, and 1.55% at 1, 3, 7, 28, and 90 days, respectively, compared to the control mix (NC0.0). This enhancement reflects the continuous progression of cement hydration and the beneficial role of nano-CaCO₃, which promotes hydration reactions and pozzolanic interactions with calcium hydroxide (Ca(OH)₂), leading to the formation of additional cementitious products. Furthermore, nano-CaCO₃ particles interact with C₃A (tricalcium aluminate) to form carboaluminate phases. These compounds possess a distinct structure characterized by strong hydrogen bonds between oxygen atoms and interlayer water molecules in CO₃²⁻ groups, which contribute to improved matrix strength and a refined microstructure [63].

The findings also show that Wn (%) values generally increase with hydration time but decrease with higher nano-CaCO₃ content (in mixes NC1.0 and NC1.5). This decline may be attributed to the dilution effect, where cement is partially replaced by an inert filler, and the agglomeration of nanoparticles within the slurry and cement matrix. As a result, the Wn values of NC1.0 and NC1.5 composites were reduced by 5.27% and 7.48%, respectively, at 90 days compared to the control mix (NC0.0), indicating a less effective hydration process.

#### X-ray diffraction

**Fig. 16:**
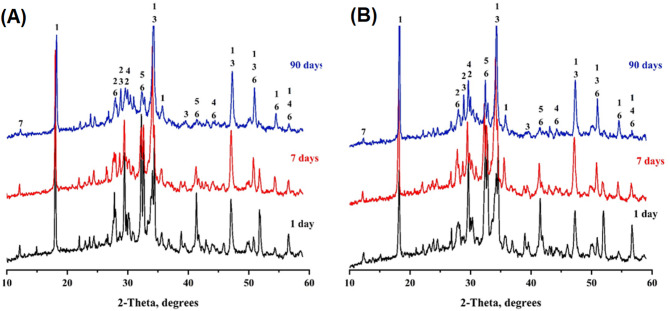
XRD-patterns for the selected composite pastes NC0.0 (A) and NC0.5 (B) at 1, 7 and 90 days of curing, 1 = CH, 2 = CSH, 3 = CASH, 4= $\:\text{C}\stackrel{-}{\text{C}}$, 5 = β-C_2_S, 6=C_3_S, 7= Ettringite

Figure 16 presents the X-ray diffraction (XRD) patterns of NC0.0 (control) and NC0.5 composite pastes cured at 1, 7, and 90 days. The XRD profiles of NC0.0 confirm the formation of key hydration products, as evidenced by sharp crystalline peaks corresponding to calcium hydroxide (CH, Ca(OH)₂) at 2θ = 18.05°, 34.38°, 36.75°, 47.05°, and 50.75°. Broad, ill-defined peaks observed at 2θ = 29.43°, 34.38°, and 39.35° are attributed to poorly crystalline calcium silicate hydrate (C-S-H) and calcium aluminosilicate hydrate (C-A-S-H) phases. Additionally, peaks assigned to unreacted silicate phases such as alite (3CaO·SiO₂) and belite (2CaO·SiO₂) were identified at 2θ = 29.43°, 32.07°, 41.68°, 43.14°, and 51.76°. Over time, some of the CH formed during hydration underwent carbonation, giving rise to identifiable peaks for calcite (CaCO₃).

As shown in Fig. 16A, the intensities of C-S-H, C-A-S-H, and CH peaks increased with hydration time, reaching their highest values at 90 days, indicative of progressive hydration. In contrast, the intensity of peaks corresponding to unhydrated alite and belite decreased, which aligns with the expected consumption of these phases during the hydration process. Figure 16B shows the XRD pattern for the NC0.5 composite paste, which exhibits the same hydration phases as the control (NC0.0), but with notably higher peak intensities, particularly for CH at 2θ = 18.05°. This suggests an enhancement in the degree of crystallinity due to the presence of nano-CaCO₃. The reduced intensities of alite and belite peaks further indicate their accelerated transformation into hydration products such as C-S-H and C-A-S-H. These observations confirm the catalytic role of nano-CaCO₃ particles (with a specific surface area of 48.455 m²/g and an average particle size of 2.269 nm) in promoting early and more extensive hydration, thereby increasing the quantity of hydration products.

#### Thermal analysis

**Fig. 17:**
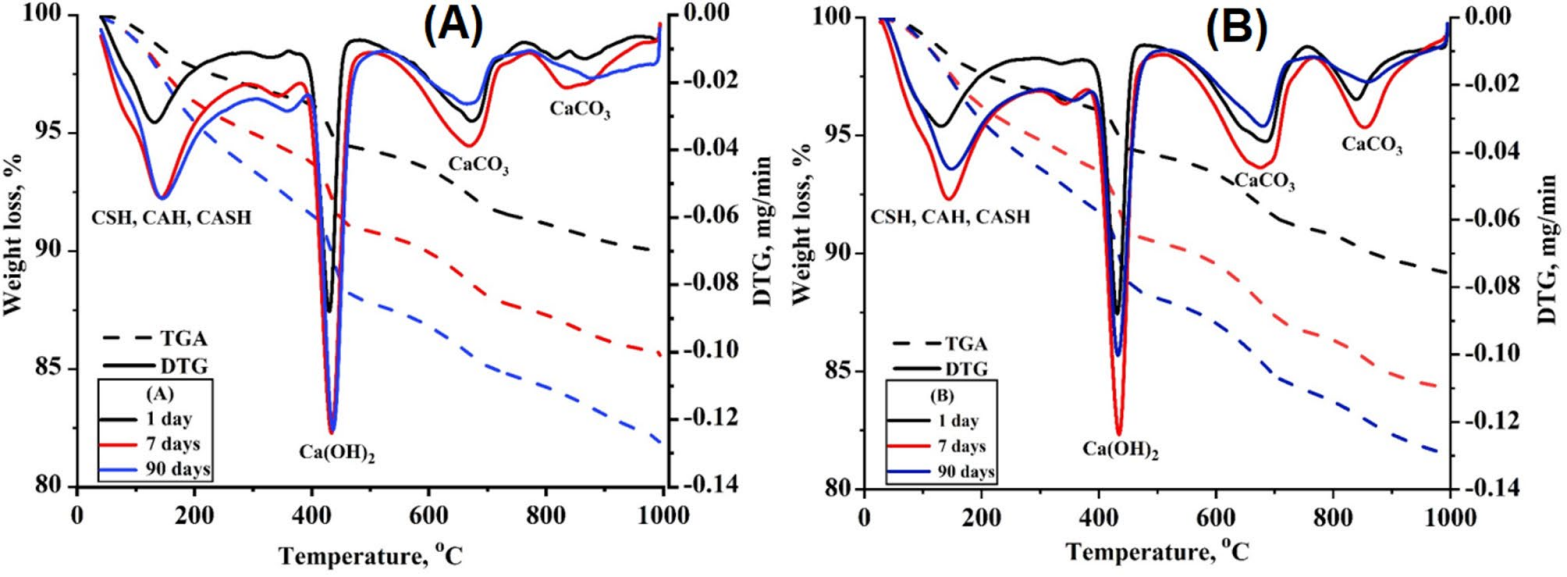
DTGA/TGA for the selected composite pastes NC0.0 (A) and NC0.5 (B) at 1, 7, and 90-days of curing

Figure 17 presents the differential thermogravimetric analysis (DTG/TGA) thermograms of composite pastes NC0.0 and NC0.5 after 1, 7, and 90 days of hydration. In general, four distinct endothermic peaks are identified within the temperature ranges of 90–210 °C, 400–450 °C, 600–650 °C, and 800–950 °C. The first peak, observed between 90 and 210 °C, corresponds to the loss of physically adsorbed water and the thermal decomposition or dehydration of amorphous hydration products such as calcium silicate hydrate (C-S-H), calcium aluminosilicate hydrate (C-A-S-H), and calcium aluminate hydrate (C-A-H) [64].

The TGA weight loss percentages for NC0.0 and NC0.5 at 1 day of hydration were recorded as 2.42% and 3.01%, respectively. After 7 days, these values increased to 4.22% for NC0.0 and 4.02% for NC0.5. At 90 days, the weight loss further increased to 4.77% and 4.85% for NC0.0 and NC0.5, respectively. These results indicate a progressive increase in hydration product formation over time and confirm the positive influence of nano-CaCO₃ (NC) addition. The replacement of OPC with 0.5% NC promoted the formation of additional hydration products such as C-S-H, C-A-S-H, and C-A-H. The second endothermic peak, observed between 400 and 450 °C, is attributed to the thermal decomposition of calcium hydroxide (CH). The associated weight loss was 2.19% and 2.07% for NC0.0 and NC0.5, respectively, at 1 day. After 7 days, these values increased to 3.22% (NC0.0) and 3.28% (NC0.5), and further to 4.23% and 3.86% after 90 days. These findings suggest that substituting OPC with nano-CaCO₃ reduced the accumulation of CH, especially over longer curing periods. The endothermic peaks observed at 600–650 °C and 800–950 °C correspond to the thermal decomposition of calcium carbonate (CaCO₃) with varying degrees of crystallinity. Notably, the weight loss associated with these peaks in NC0.5 was higher than in NC0.0, indicating a greater presence of carbonate phases. This result can be attributed to the catalytic role of NC nanoparticles, which not only promote additional hydration reactions but also lead to enhanced formation of carbonate-containing phases.

#### Morphology

**Fig. 18:**
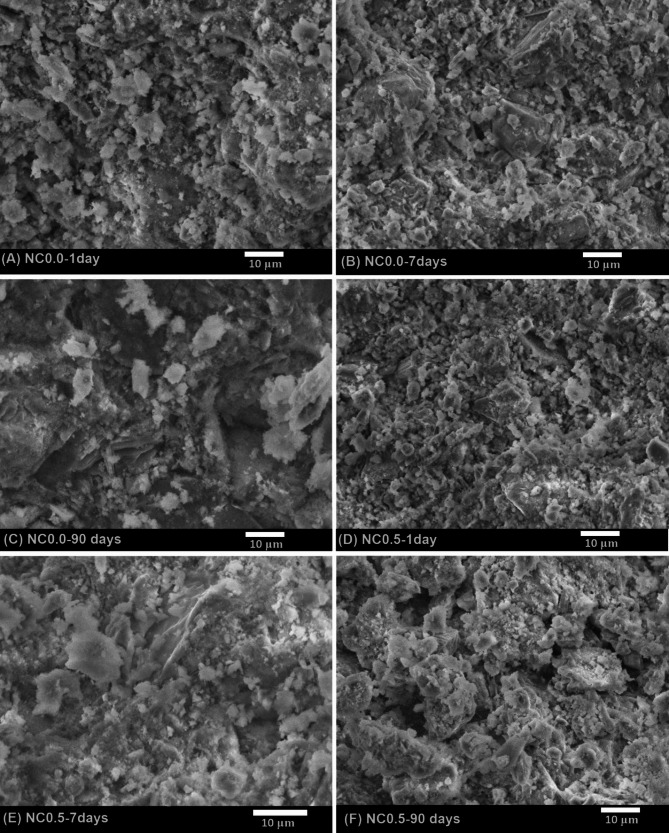
SEM images of NC0.0 and NC0.5 cement composites; (A) NC0.0-1 day; (B) NC0.0-7 days; (C) NC0.0-90 days; (D) NC0.5-1 day; (E) NC0.5-7 days; and (E) NC0.5-90 days

The Scanning Electron Microscopy (SEM) technique was employed to examine the morphological characteristics of the hydration products, which closely correlate with the mechanical properties of the hardened cement paste [65]. Figure 18A-F presents the SEM micrographs of the NC0.0 and NC0.5 cement composites, respectively, after hydration at three different intervals upto 90 days.

At 1 day of hydration, the microstructure of the NC0.0 paste displays the initial formation of small quantities of amorphous and microcrystalline calcium silicate hydrate (C–S–H) like gel, along with hexagonal crystals of calcium hydroxide (CH) and minor amounts of fibrous ettringite (AFt) dispersed within the matrix (Fig. 18A). After 7 and 90 days of curing, the micrographs of the NC0.0 mix reveal a progressively denser microstructure characterized by the presence of abundant needle-shaped AFt fibers and tobermorite-like C–S–H crystals intermixed with hexagonal CH crystals (Figs. 18B, C). Additionally, small quantities of calcium carbonate (C$\:\stackrel{-}{\text{C}}$) are observed, attributed to partial carbonation during sample handling. Voids remain within the matrix, suggesting potential for further hydration.

The SEM micrographs of the NC0.5 cement composite hydrated at 1 and 7 days (Figs. 18D, E) confirm the formation of a considerable amount of needle-shaped C–S–H, AFt fibers, and CH crystals. The increased presence of C–S–H in the NC0.5 mix can be attributed to the accelerating effect of the CaCO₃-nanoparticles, which enhance the nucleation and growth of hydration products. After 90 days of curing, the microstructure exhibits a densely packed morphology featuring rod-like C–S–H crystals interspersed with thin plates and stacked sheets of hexagonal CH and calcium aluminum silicate hydrate (C–A–S–H) phases, along with a minor presence of CaCO₃ (Fig. 18F).

### Conclusion

This study successfully demonstrated the potential of *Bacillus tropicus* strain D16 in the biomineralization of calcium carbonate (CaCO₃), highlighting its viability as a sustainable alternative to conventional cement in construction materials. The isolate exhibited a high capacity for CaCO₃ precipitation under optimal conditions. The properties of the precipitated CaCO₃ were confirmed using SEM equipped with EDX analysis, FT-IR spectroscopy, and XRD. The findings suggest that *Bacillus tropicus* could serve as a bio-based cement, contributing to reduced environmental impacts associated with traditional cement production and usage. Four cementitious composite pastes were prepared using varying dosages of CaCO₃ nanoparticles (NPs): NC0.0, NC0.5, NC1.0, and NC1.5. These pastes were hydrated for upto 90 days under high humidity conditions at 25 °C. The effects of nano-CaCO₃ on compressive strength, bulk density, total porosity, and chemically combined water content were systematically investigated. The key findings are summarized as follows:

1. The incorporation of 0.5 wt% CaCO₃ nanoparticles significantly enhanced the compressive strength of the control paste (NC0.0) throughout the 90-day curing period. However, increasing the dosage to 1.0 wt% or higher resulted in a reduction in strength, primarily due to nanoparticle agglomeration and their high surface energy, which hinders uniform dispersion.
2. Both chemically combined water content (Wₙ) and bulk density showed notable improvements with the addition of up to 0.5 wt% CaCO₃ NPs. Beyond this concentration (i.e., at 1.0 wt% and above), these parameters declined, indicating a dilution effect and potential microstructural disruption due to nanoparticle agglomeration.
3. Characterization techniques, including X-ray diffraction (XRD), scanning electron microscopy (SEM), and thermogravimetric analysis (TGA/DTG) confirmed the catalytic activity of CaCO₃ nanoparticles within the cementitious matrix. These nanoparticles promoted the formation of additional strength-contributing hydration products such as calcium silicate hydrates (C-S-H), calcium aluminosilicate hydrates (C-A-S-H), and calcium aluminate hydrates (C-A-H).

## Data Availability

No datasets were generated or analysed during the current study.

## References

[1] Granger S, Loukili A, Pijaudier-Cabot G, Chanvillard G. Experimental characterization of the self-healing of cracks in an ultra high performance cementitious material: mechanical tests and acoustic emission analysis. Cem Concr Res. 2007;37(4):519–27.

[2] Mignon A, Snoeck D, Dubruel P, Van Vlierberghe S, De Belie N. Crack mitigation in concrete: superabsorbent polymers as key to success? Materials. 2017;10(3):237. 28772599 10.3390/ma10030237 PMC5503349

[3] Chahal N, Siddique R, Rajor A. Influence of bacteria on the compressive strength, water absorption and rapid chloride permeability of fly Ash concrete. Constr Build Mater. 2012;28(1):351–6.

[4] Vijay K, Murmu M, Deo SV. Bacteria based self healing concrete–A review. Constr Build Mater. 2017;152:1008–14.

[5] Javeed Y, Goh Y, Mo KH, Yap SP, Leo BF. Microbial self-healing in concrete: A comprehensive exploration of bacterial viability, implementation techniques, and mechanical properties. J Mater Res Technol. 2024;29:2376–95.

[6] Jonkers HM. Bacteria-based self-healing concrete. Heron. 2011;56(1/2):1–12.

[7] Jena S, Basa B, Panda KC, Sahoo NK. *Impact of Bacillus subtilis bacterium on the properties of concrete.* Materials Today: Proceedings, 2020. 32: pp. 651–656.

[8] de Brito J, Kurda R. The past and future of sustainable concrete: A critical review and new strategies on cement-based materials. J Clean Prod. 2021;281:123558.

[9] Khushnood RA, Qureshi ZA, Shaheen N, Ali S. Bio-mineralized self-healing recycled aggregate concrete for sustainable infrastructure. Sci Total Environ. 2020;703:135007. 31744694 10.1016/j.scitotenv.2019.135007

[10] Wang XF, Yang ZH, Fang C, Han NX, Zhu GM, Tang JN, Xing F. Evaluation of the mechanical performance recovery of self-healing cementitious materials–its methods and future development: a review. Constr Build Mater. 2019;212:400–21.

[11] Van Tittelboom K, De Belie N. Self-healing in cementitious materials—A review. Materials. 2013;6(6):2182–217. 28809268 10.3390/ma6062182 PMC5458958

[12] Huang H, Ye G, Qian C, Schlangen E. (2016). Self-healing in cementitious materials: Materials & Design, 2016. 92: pp. 499–511.

[13] Bundur ZB, Kirisits MJ, Ferron RD. Use of pre-wetted lightweight fine expanded shale aggregates as internal nutrient reservoirs for microorganisms in bio-mineralized mortar. Cem Concr Compos. 2017;84:167–74.

[14] Jonkers HM, Thijssen A, Muyzer G, Copuroglu O, Schlangen E. Application of bacteria as self-healing agent for the development of sustainable concrete. Ecol Eng. 2010;36(2):230–5.

[15] Vijay K, Murmu M. Effect of calcium lactate on compressive strength and self-healing of cracks in microbial concrete. Front Struct Civil Eng. 2019;13:515–25.

[16] Pei R, Liu J, Wang S, Yang M. Use of bacterial cell walls to improve the mechanical performance of concrete. Cem Concr Compos. 2013;39:122–30.

[17] Wang JY, Soens H, Verstraete W, De Belie N. Self-healing concrete by use of microencapsulated bacterial spores. Cem Concr Res. 2014;56:139–52.

[18] Nuaklong P, Jongvivatsakul P, Phanupornprapong V, Intarasoontron J, Shahzadi H, Pungrasmi W, Likitlersuang S. Self-repairing of shrinkage crack in mortar containing microencapsulated bacterial spores. J Mater Res Technol. 2023;23:3441–54.

[19] Wu M, Hu X, Zhang Q, Xue D, Zhao Y. Growth environment optimization for inducing bacterial mineralization and its application in concrete healing. Constr Build Mater. 2019;209:631–43.

[20] Tayebani B, Mostofinejad D. Penetrability, corrosion potential, and electrical resistivity of bacterial concrete. J Mater Civ Eng. 2019;31(3):04019002.

[21] Priya TS, Ramesh N, Agarwal A, Bhusnur S, Chaudhary K. Strength and durability characteristics of concrete made by micronized biomass silica and Bacteria-Bacillus sphaericus. Constr Build Mater. 2019;226:827–38.

[22] Espitia-Nery ME, Corredor-Pulido DE, Castaño-Oliveros PA, Rodríguez-Medina JA, Ordoñez-Bello QY, Pérez-Fuentes MS. Mechanisms of encapsulation of bacteria in self-healing concrete. Dyna. 2019;86(210):17–22.

[23] Schwantes-Cezario N, Porto MF, Sandoval GFB, Nogueira GFN, Couto AF, Toralles BM. Effects of Bacillus subtilis biocementation on the mechanical properties of mortars. Revista IBRACON De Estruturas E Materiais. 2019;12(01):31–8.

[24] Durga CSS, Ruben N, Chand MSR, Venkatesh C. Performance studies on rate of self healing in bio concrete. Mater Today: Proc. 2020;27:158–62.

[25] Eltarahony M, Zaki S, Abd-El-Haleem D. Aerobic and anaerobic removal of lead and mercury via calcium carbonate precipitation mediated by statistically optimized nitrate reductases. Sci Rep. 2020;10(1):4029. 32132620 10.1038/s41598-020-60951-1 PMC7055279

[26] Awadeen NA, Eltarahony M, Zaki S, Yousef A, El-Assar S, El-Shall H. Fungal carbonatogenesis process mediates zinc and chromium removal via statistically optimized carbonic anhydrase enzyme. Microb Cell Fact. 2024;23(1):236. 39192338 10.1186/s12934-024-02499-7 PMC11350955

[27] Eltarahony M, Kamal A, Zaki S, Abd-El‐Haleem D. Heavy metals bioremediation and water softening using ureolytic strains Metschnikowia pulcherrima and Raoultella planticola. J Chem Technol Biotechnol. 2021;96(11):3152–65.

[28] Krajewska B. Urease-aided calcium carbonate mineralization for engineering applications: A review. J Adv Res. 2018;13:59–67. 30094083 10.1016/j.jare.2017.10.009 PMC6077181

[29] O’connell M, McNally C, Richardson MG. Biochemical attack on concrete in wastewater applications: A state of the Art review. Cem Concr Compos. 2010;32(7):479–85.

[30] Cao M, Ming X, He K, Li L, Shen S. Effect of macro-, micro-and nano-calcium carbonate on properties of cementitious composites—A review. Materials. 2019;12(5):781. 30866439 10.3390/ma12050781 PMC6427187

[31] Sobolev K. Modern developments related to nanotechnology and nanoengineering of concrete. Front Struct Civil Eng. 2016;10:131–41.

[32] Cosentino I, Restuccia L, Ferro GA, Liendo F, Deorsola F, Bensaid S. *Evaluation of the mechanical properties of cements with fillers derived from the CO*_*2*_*reduction of cement plants*. Procedia Struct Integr. 2019;18:472–83.

[33] Sun Y, Zhang P, Guo W, Bao J, Qu C. *Effect of Nano-CaCO*_*3*_*on the Mechanical Properties and Durability of Concrete Incorporating Fly Ash*. Adv Mater Sci Eng. 2020;2020(1):7365862.

[34] Ekprasert J, Fongkaew I, Chainakun P, Kamngam R, Boonsuan W. *Investigating mechanical properties and biocement application of CaCO*_*3*_*precipitated by a newly-isolated Lysinibacillus sp. WH using artificial neural networks*. Sci Rep. 2020;10(1):16137. 32999379 10.1038/s41598-020-73217-7 PMC7527966

[35] Loewen PC, Switala J, Triggs-Raine BL. Catalases HPI and HPII in Escherichia coli are induced independently. Arch Biochem Biophys. 1985;243(1):144–9. 3904630 10.1016/0003-9861(85)90782-9

[36] Algaifi HA, Bakar SA, Sam ARM, Ismail M, Abidin ARZ, Shahir S, Altowayti WAH. Insight into the role of microbial calcium carbonate and the factors involved in self-healing concrete. Constr Build Mater. 2020;254:119258.

[37] Ankolekar C, Rahmati T, Labbé RG. Detection of toxigenic Bacillus cereus and Bacillus Thuringiensis spores in US rice. Int J Food Microbiol. 2009;128(3):460–6. 19027973 10.1016/j.ijfoodmicro.2008.10.006

[38] Silva FV. Use of power ultrasound to enhance the thermal inactivation of clostridium perfringens spores in beef slurry. Int J Food Microbiol. 2015;206:17–23. 25912313 10.1016/j.ijfoodmicro.2015.04.013

[39] Lv, R., Zou, M., Chantapakul, T., Chen, W., Muhammad, A. I., Zhou, J., … Liu, D.*Effect of ultrasonication and thermal and pressure treatments, individually and combined,on inactivation of Bacillus cereus spores.* Applied microbiology and biotechnology, 2019. 103: pp. 2329–2338. 10.1007/s00253-018-9559-3 30627794

[40] Pandey R, MuÈller A, Napoli CA, Selinger DA, Pikaard CS, Richards EJ, Jorgensen RA. Analysis of histone acetyltransferase and histone deacetylase families of Arabidopsis Thaliana suggests functional diversification of chromatin modification among multicellular eukaryotes. Nucleic Acids Res. 2002;30(23):5036–55. 12466527 10.1093/nar/gkf660 PMC137973

[41] Al Qabany A, Soga K, Santamarina C. Factors affecting efficiency of microbially induced calcite precipitation. J Geotech GeoEnviron Eng. 2012;138(8):992–1001.

[42] Abou-Taleb KA, Sayed LM, Abou-Taleb K. Utilization of Agro-industrial residues for production of amino acids using Egyptian Bacillus spp. Strains. Middle East J Appl Sci. 2012; 2(2):71-82.

[43] Heikal M, Nassar MY, Abd El-ALeem S, El.Sharkawy AM, El-Hassan D, Ibrahim SM. Performance of composite cement containing nano -ZnO subjected to elevated. Benha J Appl Sci. 2022;7:115–30.

[44] Stocks-Fischer S, Galinat JK, Bang SS. Microbiological precipitation of CaCO3. Soil Biol Biochem. 1999;31(11):1563–71.

[45] Danish A, Mosaberpanah MA, Salim MU. Past and present techniques of self-healing in cementitious materials: A critical review on efficiency of implemented treatments. J Mater Res Technol. 2020;9(3):6883–99.

[46] Higgins D, Dworkin J. Recent progress in Bacillus subtilis sporulation. FEMS Microbiol Rev. 2012;36(1):131–48. 22091839 10.1111/j.1574-6976.2011.00310.x PMC3237856

[47] Carrera M, Zandomeni RO, Fitzgibbon J, Sagripanti JL. Difference between the spore sizes of Bacillus anthracis and other Bacillus species. J Appl Microbiol. 2007;102(2):303–12. 17241334 10.1111/j.1365-2672.2006.03111.x

[48] Perez-Valdespino A, Ghosh S, Cammett EP, Kong L, Li YQ, Setlow P. *Isolation and characterization of Bacillus subtilis spores that are superdormant for germination with dodecylamine or Ca*^*2+*^*‐dipicolinic acid*. J Appl Microbiol. 2013;114(4):1109–19. 23289722 10.1111/jam.12125

[49] Erdmann N, Strieth D. Influencing factors on ureolytic microbiologically induced calcium carbonate precipitation for biocementation. World J Microbiol Biotechnol. 2023;39(2):61. 10.1007/s11274-022-03499-8 PMC9797461 36576609

[50] Wang J, Van Tittelboom K, De Belie N, Verstraete W. Use of silica gel or polyurethane immobilized bacteria for self-healing concrete. Constr Build Mater. 2012;26(1):532–40.

[51] Nguyễn HH, Choi JI, Park SE, Cha SL, Huh J, Lee BY. Autogenous healing of high strength engineered cementitious composites (ECC) using calcium-containing binders. Constr Build Mater. 2020;265:120857.

[52] Choudhary A, Onuaguluchi O, Banthia N. Exogenous healing in concrete with pH-sustained internal carbonation. Cem Concr Compos. 2021;123:104173.

[53] Wei S, Cui H, Jiang Z, Liu H, He H, Fang N. Biomineralization processes of calcite induced by bacteria isolated from marine sediments. Brazilian J Microbiol. 2015;46:455–64. 10.1590/S1517-838246220140533 PMC4507537 26273260

[54] Gu Z, Chen Q, Wang L, Niu S, Zheng J, Yang M, Yan Y. Morphological changes of calcium carbonate and mechanical properties of samples during microbially induced carbonate precipitation (MICP). Materials. 2022;15(21):7754. 36363345 10.3390/ma15217754 PMC9655993

[55] Frankel RB, Bazylinski DA. Biologically induced mineralization by bacteria. Rev Mineral Geochem. 2003;54(1):95–114.

[56] Ramasamy V, Anand P, Suresh G. *Biomimetic Synthesis and characterization of precipitated CaCO*_*3*_*nanoparticles using different natural carbonate sources: A novel approach*. Int J Mater Sci. 2017;12:499–511.

[57] Qi S, Xue Q, Niu Z, Zhang Y, Liu F, Chen H. Inve*stigation of Zn*^*2+*^*and Cd*^*2+*^*adsorption performance by different weathering basalts*. Water Air Soil Pollut. 2016;227:1–11.

[58] Muhsin ZF, Fawzi NM. *Effect of nano calcium Carbonate on some properties of reactive powder concrete*. in *IOP Conference Series: Earth and Environmental Science*. 2021. IOP Publishing.

[59] Supit SW, Shaikh FU. *Effect of nano-CaCO*_*3*_*on compressive strength development of high volume fly ash mortars and concretes*. J Adv Concr Technol. 2014;12(6):178–86.

[60] Moon J, Oh JE, Balonis M, Glasser FP, Clark SM, Monteiro PJ. *High pressure study of low compressibility tetracalcium aluminum carbonate hydrates 3CaO· Al*_*2*_*O*_*3*_*· CaCO*_*3*_*· 11H*_*2*_*O*. Cem Concr Res. 2012;42(1):105–10.

[61] Damidot D, Stronach S, Kindness A, Atkins M, Glasser FP. *Thermodynamic investigation of the CaO Al*_*2*_*O*_*3*_*CaCO*_*3*_*H*_*2*_*O closed system at 25° C and the influence of Na*_*2*_*O*. Cem Concr Res. 1994;24(3):563–72.

[62] Liu X, Chen L, Liu A, Wang X. E*ffect of nano-CaCO3 on properties of cement paste*. Energy Procedia. 2012;16:991–6.

[63] Alyousef R, Benjeddou O, Soussi C, Khadimallah MA, Mustafa Mohamed A. Effects of incorporation of marble powder obtained by recycling waste sludge and limestone powder on rheology, compressive strength, and durability of Self-Compacting concrete. Adv Mater Sci Eng. 2019;2019(1):4609353.

[64] Ibrahim SM, Heikal M, Abdelwahab NR, Mohamed OA. *Fabricated CeO*_*2*_*/ZrO*_*2*_*nanocomposite to improve thermal resistance, mechanical characteristics, microstructure and gamma radiation shielding of OPC composite cement pastes*. Constr Build Mater. 2023;392:131971.

[65] Abdel-Monem DM, Asker MS, Ali EE, Abdel-Monem MO. Biomineralization of CaCO. Bacillus Sp 8WNM Application as Bio-Cement J Basic Environ Sci. 2024;11(4):326–40.

